# Supplementary material for: Universal machine learning aided synthesis approach of two-dimensional perovskites in a typical laboratory
Source: Nat Commun. 2024 Jan 2;15:138. doi: 10.1038/s41467-023-44236-5 (PMC10761762; doi:10.1038/s41467-023-44236-5)
Supplement: Supplementary file 1 — Supplementary Information [file 41467_2023_44236_MOESM1_ESM.pdf]

Supplementary Information

Universal machine learning aided synthesis approach of two-dimensional perovskites in a typical laboratory

Yilei Wu[1†], Chang-Feng Wang[2†], Ming-Gang Ju[1†]*, Qiangqiang Jia[2], Qionghua Zhou[1], Shuaihua Lu[1], Xinying Gao[1], Yi Zhang[2]*, and Jinlan Wang[1,3]*

[1] Key Laboratory of Quantum Materials and Devices of Ministry of Education, School of Physics, Southeast University, Nanjing 211189, China.

[2]Institute for Science and Applications of Molecular Ferroelectrics, Key Laboratory of the Ministry of Education for Advanced Catalysis Materials, Zhejiang Normal University, Jinhua, 321004, China

[3]Suzhou Laboratory, Suzhou, China

[†]These authors contributed equally: Yilei Wu, Chang-Feng Wang, and Ming-Gang Ju

[*]E-mail: juming@seu.edu.cn (M.-G. J.); yizhang1980@seu.edu.cn (Y. Z.); jlwang@seu.edu.cn (J. W.)

Supplementary Methods

Synthesis methods.

Compounds in high-throughput synthesis experiments were prepared by utilizing evaporation method, the synthetic chemical reagents are reagent grade and are not further purified when used. It is worth noting that an excess amount of Ag_2CO_3 was used to eliminate the competing Bi-based phases. To assess the experimental reproducibility of our synthesis experiments, we conducted ten individual repetitions of the synthesis process for $(NH_2C_5H_8F_2)_4AgBiI_8$. Remarkably, in each instance, we successfully synthesized 2D perovskites (Supplementary Fig 3), indicating excellent reproducibility. The detailed synthesis processes of successfully synthesized 2D AgBi perovskites in training set are as follows.

$(ClC_6H_4CH_4NH_3)_4AgBiI_8$: An amount of 1.5 mmol Ag_2CO_3 (413.6 mg) and 0.25 mmol Bi_2O_3 (116.5 mg) were dissolved in 5 mL concentrated hydroiodic acid (HI, 55.0-58.0%) under heating at 393 K and stirred for 10 min. 2 mmol (*S*)-1-(4-chlorophenyl)ethan-1-amine (311.2 mg) was added to 1 mL H_3PO_2 in a separate beaker. Then two cups of the solution were mixed. The mixing solution was allowed to evaporate at the hot plate with 323 K. After a day, brownish red crystals precipitated at the bottom of the beaker.

$(BrC_6H_4CH_4NH_3)_4AgBiI_8$: An amount of 1.5 mmol Ag_2CO_3 (413.6 mg) and 0.25 mmol Bi_2O_3 (116.5 mg) were dissolved in 8 mL HI and 1 mL H_3PO_2 under heating at 393 K and stirred for 10 min. 2 mmol (*S*)-1-(4-bromophenyl)ethan-1-amine (400 mg) was added to 1 mL H_3PO_2 and 0.5 ml HI in a separate beaker. Then mix cups of the solution were mixed. The mixing solution was allowed to evaporate at the hot plate with 323 K. After a day, brownish red crystals precipitated at the bottom of the beaker.

$(CH_3C_6H_4C_2H_4NH_3)_4AgBiI_8$: An amount of 1 mmol Ag_2CO_3 (275.7 mg) and 0.25 mmol Bi_2O_3 (116.5 mg) were dissolved in 5 mL HI and 0.5 mL H_3PO_2 under heating at 393 K and stirred for 10 min. 2 mmol (*S*)-1-(*p*-tolyl)ethan-1-amine (270.4 mg) was added to 1 mL H_3PO_2 in a separate beaker. Then two cups of the solution were mixed. The mixing solution was allowed to evaporate at the hot plate with 323 K. After two days, brownish red crystals precipitated at the bottom of the beaker.

$(ClC_6H_4NH_3)_4AgBiI_8$: An amount of 1.5 mmol Ag_2CO_3 (413.6 mg) and 0.25 mmol

Bi_2O_3 (116.5 mg) were dissolved in 6 mL HI under heating at 393 K and stirred for 10 min. 2 mmol 4-chloroaniline (255.1 mg) was added to 1 mL H_3PO_2 and 1 ml HI in a separate beaker. Then two cups of the solution were mixed. The mixing solution was allowed to evaporate at the hot plate with 343 K. After a day, brownish red crystals precipitated at the bottom of the beaker.

$(NH_3C_6H_{10}NH_3)_2AgBiI_8 \cdot H_2O$: An amount of 1 mmol Ag_2CO_3 (275.7 mg) and 0.25 mmol Bi_2O_3 (116.5 mg) were dissolved in 5 mL HI and 0.5 mL H_3PO_2 under heating at 393 K and stirred for 10 min. 1 mmol cyclohexane-1,4-diamine (114.1 mg) was added to 1 mL H_3PO_2 in a separate beaker. Then two cups of the solution were mixed. The mixing solution was allowed to evaporate at the hot plate with 323 K. After a day, brownish red crystals precipitated at the bottom of the beaker.

$(NH_2C_5H_9CH_2NH_3)_2AgBiI_8 \cdot 0.5H_2O$: An amount of 1 mmol Ag_2CO_3 (275.7 mg) and 0.25 mmol Bi_2O_3 (116.5 mg) were dissolved in 5 mL HI under heating at 393 K and stirred for 10 min. 1 mmol 4-(aminomethyl)piperidine (114.1 mg) was added to 1 mL H_3PO_2 in a separate beaker. Then two cups of the solution were mixed. The mixing solution was allowed to evaporate at the hot plate with 323 K. After a day, brownish red crystals precipitated at the bottom of the beaker.

$(NH_2C_4H_6F_2)_4AgBiI_8 \cdot H_2O$: An amount of 1 mmol Ag_2CO_3 (275.7 mg) and 0.25 mmol Bi_2O_3 (116.5 mg) were dissolved in 5 mL HI under heating at 393 K and stirred for 10 min. 2 mmol 3,3-difluoropyrrolidine hydrochloride (287 mg) was added to 1 mL H_3PO_2 in a separate beaker. Then two cups of the solution were mixed. The mixing solution was allowed to evaporate at the hot plate with 323 K. After a day, red crystals precipitated at the bottom of the beaker.

$(IC_3H_6NH_3)_4AgBiI_8$: An amount of 1 mmol Ag_2CO_3 (275.7 mg) and 0.25 mmol Bi_2O_3 (116.5 mg) were dissolved in 5 mL HI and 1 mL H_3PO_2 under heating at 393 K and stirred for 10 min. 2 mmol 3-methoxypropylamine (178.2 mg) was added to 1 mL HI in a separate beaker under heating at 343 K and stirred for 10 min. Then two cups of the solution were mixed. The mixing solution was allowed to evaporate at the hot plate with 313 K. After a day, black crystals precipitated at the bottom of the beaker.

$(ClFC_6H_3NH_3)_4AgBiI_8$: An amount of 1 mmol Ag_2CO_3 (275.7 mg) and 0.25 mmol

Bi_2O_3 (116.5 mg) were dissolved in 5 mL HI under heating at 393 K and stirred for 10 min. 2 mmol 3-fluoro-4-chloroaniline (291.1 mg) was added to 1 mL H_3PO_2 in a separate beaker. Then two cups of the solution were mixed. The mixing solution was allowed to evaporate at the hot plate with 343 K. After a day, brownish red crystals precipitated at the bottom of the beaker.

$(NH_2C_5H_9F)_4AgBiI_8 \cdot H_2O$: An amount of 1 mmol Ag_2CO_3 (275.7 mg) and 0.25 mmol Bi_2O_3 (116.5 mg) were dissolved in 5 mL HI under heating at 393 K and stirred for 10 min. 2 mmol 3-fluoropiperidine hydrochloride (280 mg) was added to 1 mL H_3PO_2 in a separate beaker. Then two cups of the solution were mixed. The mixing solution was allowed to evaporate at the hot plate with 343 K. After a day, brownish red crystals precipitated at the bottom of the beaker.

$(NH_2C_5H_8F_2)_4AgBiI_8$: An amount of 0.5 mmol Ag_2CO_3 (137.8 mg) and 1 mmol $BiCl_3$ (315.3 mg) were dissolved in 10 mL HI under heating at 373 K and stirred for 10 min. 4 mmol 4,4-difluoropiperidinehydrochloride (630.36 mg) was added to 1 mL H_3PO_2 and 1 mL HI in a separate beaker. Then two cups of the solution were mixed. The mixing solution was allowed to evaporate at the hot plate with 343 K. After two days, brownish red crystals precipitated at the bottom of the beaker.

$(N_2C_3H_4C_2H_4NH_3)_2AgBiI_8$: An amount of 2.5 mmol Ag_2CO_3 (689.2 mg) and 1 mmol $BiCl_3$ (315.3 mg) were dissolved in 10 mL HI under heating at 373 K and stirred for 10 min. 2 mmol histamine dihydrochloride (368.2 mg) was added to 1 mL H_3PO_2 and 1 mL HI in a separate beaker. Then two cups of the solution were mixed. The mixing solution was allowed to evaporate at the hot plate with 343 K. After a day, black crystals precipitated at the bottom of the beaker.

$(F_3C_4H_6NH_3)_4AgBiI_8$: An amount of 2.5 mmol Ag_2CO_3 (689.2 mg) and 1 mmol $BiCl_3$ (315.3 mg) were dissolved in 10 mL HI under heating at 373 K and stirred for 10 min. 4 mmol 4,4,4-trifluorobutan-1-amine hydrochloride (654 mg) was added to 1 mL H_3PO_2 and 2 mL HI in a separate beaker. Then two cups of the solution were mixed. The mixing solution was allowed to evaporate at the hot plate with 343 K. After a day, red crystals precipitated at the bottom of the beaker.

The detailed synthesis processes of 8 predicted 2D AgBi perovskites are as follows.

$(C_6H_{11}NH_3)_4AgBiI_8$: An amount of 1 mmol Ag_2CO_3 (275.7 mg) and 0.25 mmol Bi_2O_3 (116.5 mg) were dissolved in 8 mL HI under heating at 393 K and stirred for 10 min. 2 mmol cyclohexylamine (200 mg) was added to 1 mL H_3PO_2 in a separate beaker. Then two cups of the solution were mixed. The mixing solution was allowed to evaporate at the hot plate with 323 K. After a day, brownish red crystals precipitated at the bottom of the beaker.

$(FC_6H_4CH_2NH_3)_4AgBiI_8$: An amount of 1.5 mmol Ag_2CO_3 (413.6 mg) and 0.25 mmol Bi_2O_3 (116.5 mg) were dissolved in 5 mL HI under heating at 393 K and stirred for 10 min. 2 mmol 4-fluorobenzylamine (250.2 mg) was added to 1 mL H_3PO_2 in a separate beaker. Then two cups of the solution were mixed. The mixing solution was allowed to evaporate at the hot plate with 343 K. After a day, red crystals precipitated at the bottom of the beaker.

$(ClC_6H_4CH_2NH_3)_4AgBiI_8$: An amount of 2.5 mmol Ag_2CO_3 (689.3 mg) and 0.25 mmol Bi_2O_3 (116.5 mg) were dissolved in 6 mL HI under heating at 393 K and stirred for 10 min. 4 mmol 4-chlorobenzylamine (283.2 mg) was added to 1 mL H_3PO_2 in a separate beaker. Then two cups of the solution were mixed. The mixing solution was heated to 393 K and then cooled to room temperature with a rate of 10 K h^{-1}. After 9 h, black crystals precipitated at the bottom of the beaker.

$(BrC_6H_4CH_2NH_3)_4AgBiI_8$: An amount of 1.5 mmol Ag_2CO_3 (413.6 mg) and 0.25 mmol Bi_2O_3 (116.5 mg) were dissolved in 5 mL HI under heating at 393 K and stirred for 10 min. 2 mmol 4-bromobenzylamine (372.1 mg) was added to 1 mL H_3PO_2 in a separate beaker. Then two cups of the solution were mixed. The mixing solution was allowed to evaporate at the hot plate with 343 K. After a day, black crystals precipitated at the bottom of the beaker.

$(C_6H_5C_3H_6NH_3)_4AgBiI_8 \cdot H_2O$: An amount of 2.5 mmol Ag_2CO_3 (689.2 mg) and 1 mmol $BiCl_3$ (315.3 mg) were dissolved in 10 mL HI under heating at 373 K and stirred for 10 min. 4 mmol 2-phenylpropan-1-amine (540 mg) was added to 1 mL H_3PO_2 and 2 mL HI in a separate beaker. Then two cups of the solution were mixed. The mixing solution was allowed to evaporate at the hot plate with 343 K. After a day, red crystals precipitated at the bottom of the beaker.

$(FC_6H_4C_2H_4NH_3)_4AgBiI_8{\cdot}H_2O$: An amount of 2.5 mmol Ag_2CO_3 (689.2 mg) and 1 mmol $BiCl_3$ (315.3 mg) were dissolved in 10 mL HI under heating at 373 K and stirred for 10 min. 4 mmol 4-fluorophenethylamine (556.7 mg) was added to 1 mL H_3PO_2 and 2 mL HI in a separate beaker. Then two cups of the solution were mixed. The mixing solution was allowed to evaporate at the hot plate with 343 K. After a day, brownish red crystals precipitated at the bottom of the beaker.

$(NHC_5H_4C_2H_4NH_3)_2AgBiI_8$: An amount of 2.5 mmol Ag_2CO_3 (689.2 mg) and 1 mmol $BiCl_3$ (315.3 mg) were dissolved in 10 mL HI under heating at 373 K and stirred for 10 min. 2 mmol 4-(2-Aminoethyl)pyridine (244 mg) was added to 1 mL H_3PO_2 and 2 mL HI in a separate beaker. Then two cups of the solution were mixed. The mixing solution was allowed to evaporate at the hot plate with 343 K. After a day, black crystals precipitated at the bottom of the beaker.

$(NH_3C_6H_4CH_2NH_3)_2AgBiI_8$: An amount of 2.5 mmol Ag_2CO_3 (689.2 mg) and 1 mmol $BiCl_3$ (315.3 mg) were dissolved in 10 mL HI under heating at 373 K and stirred for 10 min. 2 mmol p-Aminobenzylamine (244 mg) was added to 1 mL H_3PO_2 and 2 mL HI in a separate beaker. Then two cups of the solution were mixed. The mixing solution was allowed to evaporate at the hot plate with 343 K. After a day, black crystals precipitated at the bottom of the beaker.

Experimental Characterization.

Crystal structure determination. Single clear crystals were used as supplied. Suitable crystals were selected and mounted on a XtaLAB Synergy R, HyPix diffractometer. The crystal was kept at room temperature during data collection. The structure was solved with the ShelXS (Sheldrick, 2008) or ShelXT (Sheldrick, 2014) solution program using direct methods and by using Olex2 1.5-alpha (Dolomanov et al., 2009) as the graphical interface.[1,2] The model was refined with ShelXL 2018/1 (Sheldrick, 2015) using full matrix least squares minimization on F^2.[3]

PXRD measurements. The Rigaku D/MAX 2000 x-ray diffractometer adopting Cu-Kα radiation (λ =0.15406 nm, 40 kV, and 40 mA) and a secondary beam graphite

monochromator were used to perform PXRD measurement. Measurement angle ranges from 5° to 50° with step size of 0.02° and scanning speed 2° min^{-1}.

UV–vis diffuse reflectance spectroscopy measurements. Ultraviolet–visible (UV–vis) diffuse reflectance spectroscopy measurements were performed at room temperature using a Shimadzu (Tokyo, Japan) UV-2600 spectrophotometer with an ISR–2600 Plus integrating sphere operating from 200 to 800 nm. The powder crystals were used for UV−vis absorption spectrum measurements. $BaSO_4$ was used as a 100% reflectance reference. The gradually decreasing absorption in the absorption curve indicates that all synthesized 2D AgBi perovskites exhibiting indirect bandgaps, thus the optical bandgap of selected perovskites is determined by fitting the variant Tauc equation for indirect bandgap: $(\mathrm{h\mu} \times \mathrm{F}(\mathrm{R}_{\infty}))^{\frac{1}{2}} = \mathrm{A}\left(\mathrm{h\mu} - E_{\mathrm{g}}\right)$, where h represents the Planck constant, μ represents the frequency of the photon, $\mathrm{F}(\mathrm{R}_{\infty})$ represents the Kubelka-Munk function, and A is a proportional constant.

Optical image acquisition. For Optical image acquisitions of single crystals Eclipse E600 POL polarizing microscope (Nikon) was employed.

Machine learning techniques.

Subgroup discovery algorithm. Subgroup discovery, a frequently used rule-learning technique, is developed to discover the region that stands out with a given target in the data. The subgroup discovery algorithm exhibits great difference between machine learning classification or regression model, since the former focuses on determining target subgroup at the very beginning and does not care about data out of subgroup, in contrast, the latter aims at identifying patterns in the distribution of whole data. Subgroup discovery is defined based on “local exception detection”, i.e., for a given database consisted of individuals with a target property of interest, the aim is finding statistically “most interesting” subgroups. The statistically “most interesting” subgroups display the most unusual distribution of individuals while being as large as

possible. To assess the interestingness of subgroups, various interestingness measures are developed from widely used statistical significance tests. In this work, the interestingness of subgroups of synthesis feasibility of 2D AgBi iodide perovskites are measured by utilizing the weighted relative accuracy (WRAcc). For binary classification task, the distribution of individuals in subgroup P can be displayed as follows:

	T	¬T	Total
P	p_P	n_P	i_P
¬P	$p_{\neg P}$	$n_{\neg P}$	$i_{\neg P}$
dataset	$p_\emptyset$	$n_\emptyset$	$i_\emptyset$

Where T and ¬T represent positive and negative class in binary classification task, respectively. The p, n, and i represent the number of positive instances, negative instances, and the overall number of instances for subgroup P, its complement ¬P, and the overall dataset, respectively. According to the above definition, the target concept of τ_P for subgroup P and $\tau_\emptyset$ for overall dataset can be derived as: $\tau_P = \frac{p_P}{i_P}$, $\tau_\emptyset = \frac{p_\emptyset}{i_\emptyset}$. WARcc trades of the size of subgroup i_P versus the target concept of subgroup τ_P and overall dataset $\tau_\emptyset$, which is defined as: WARcc = $i_P \times (\tau_P - \tau_\emptyset)$.[4] WARcc is the most wide-spread formalization for subgroup discovery, which is utilized to determine the boundary of black box in Figure 4a. The WARcc of all subgroups with y ranging from 486 pm to 550 pm and ^3k ranging from 1.01 to 1.89 is calculated (Supplementary Figure 13), and y and ^3k of the most "interesting" subgroup is ranging from 496 to 546 and from 1.07 to 1.82, respectively.

Problem-specific descriptors. The development of problem-specific descriptors is actually integrating physicochemical insights related to the specific problem at hand into ML model. For synthesis feasibility of 2D AgBi perovskites, the stacking mode of organic and inorganic components in 2D perovskites are dominated by the valence of organic spacers. For monovalent organic cations, adjacent inorganic layers are connected by double layers of organic layers, and two organic layers are bridged by

weak van der Waals interactions. Whereas adjacent inorganic layers of 2D perovskites with divalent organic cations are bridged by single layer of divalent organic spacers. Since organic and inorganic components of 2D perovskites are linked by hydrogen bonds between nitrogen atoms of organic spacers and terminal halide of inorganic framework, the valence of organic spacers can be obtained by counting the number of nitrogen atoms Num_N.

Due to the importance of chemical structure on the various target properties of molecules, quantifying the molecular structure of organic spacers needs to be systematically investigated. Graph theory is a useful tool for translating the chemical structure of materials into numerical topological indexes. By utilizing the molecular graph theory, the molecular topological structure can be extracted as a graph containing vertexes and edges, where vertexes and edges represent atoms and chemical bonds, respectively. Generally, hydrogens in molecules are ignored to emphasize the molecular skeleton. This pattern of connectivity of constituent atoms in molecules is called molecular topology, has been used for mathematical characterization of molecular structures and establishment of structure-property relationship. Material descriptors derived from molecular topology can characterize molecular structures from various aspects, for example, indices of Randic can be used to assess the degree of molecular branching, and distance matrix can reflect the topological distance between two atoms in a molecule. Based on the analysis for interaction between organic and inorganic components in perovskites, we found out three aspects of molecular topological structures related to synthesis feasibility of 2D AgBi perovskites, namely, interatomic distance, connectivity, and flexibility.

Distance matrix, a basis to investigate features related to molecular topological structure, is derived from the linkage of constituent atoms in the molecular skeleton. For a molecule with n atoms, the distance matrix can be defined as $D = [d_{ij}]_{n\times n}$, where d_{ij} is the shortest topological distance between atom i and j in molecular skeleton. Distance matrix can be used to effectively calculate numerical index about relationship among constituent atoms in molecules. Since the formation of strong hydrogen bonding between nitrogen of organic spacers and terminal halide of inorganic layers is critical

to the formation of 2D perovskite structure, the steric hindrance effect of protonated nitrogen should be considered as a key factor affecting the hydrogen bonding. The steric effect index (STEI) of nitrogen is defined as $\mathrm{STEI} = \sum_{i=1}^{n} \frac{1}{d_{\mathrm{N_i-Atom_j}}{}^3}$, where $d_{\mathrm{N_i-Atom_j}}$ is the distance between nitrogen i and the atom j in the molecular skeleton. For molecules with the number of nitrogen larger than 1, the maximum STEI among nitrogen is treated as the final value of STEI.

When organic spacers contain multiple nitrogen atoms, adjacent organic spacers might be bridged by single layer of divalent organic spacers, forming 2D DJ perovskites. Since the terminal halides of inorganic layers are connected with organic spacers at both ends, the strength of hydrogen bonds at both ends of organic spacers should be considered. Note that the strength of hydrogen bonding decreases as the distance between nitrogen atom and terminal halide increases, the optimized situation for two protonated nitrogen atoms is at each end of organic spacers. Two protonated nitrogen atoms that too close to each other might hinder the formation of hydrogen bond at one side, further hindering the formation of 2D perovskite structure. Thus, the distance between two nitrogen atoms is defined as $\mathrm{Dis_{NN}} = \frac{1}{d_{\mathrm{N}_i-\mathrm{N}_j}{}^2}$, where $d_{\mathrm{N}_i-\mathrm{N}_j}$ represents the distance in distance matrix between the nitrogen i and j. For molecules with one nitrogen atom, the value of $d_{\mathrm{N}_i-\mathrm{N}_j}$ is infinite, yielding the $\mathrm{Dis_{NN}}$ of 0. For molecules with the number of nitrogen atoms larger than 2, the maximum of distance between the nitrogen atom in alkyl tail and other nitrogen atoms is treated as $d_{\mathrm{N}_i-\mathrm{N}_j}$ to calculate $\mathrm{Dis_{NN}}$.

The interaction among molecules is influenced by molecular size as well as the degree of molecular branching.[5] From a molecular topology perspective, molecules with fewer branches show larger eccentricity, which is defined as the farthest path length from a vertex at the end to others in the molecular graph. Owing to the significance of hydrogen bonds, eccentricity in this work is defined as the maximum value in the row of nitrogen in the distance matrix. Since the nitrogen is always located at the end of organic spacers, eccentricity can reflect the length of molecules to some

extent. Previous study revealed the significant contribution of eccentricity of organic spacers to the formability of 2D Pb perovskites, implying organic spacers with less branches are favored to stabilize 2D perovskite structures.[6]

Another basic problem is conformational flexibility, which is highly related to the rotation and motion of organic spacers in the perovskite lattice. The conformational flexibility can be divided into two concepts, the shape of central part of organic spacers and the rotational degree of the alkyl tail. In the first case, organic spacers can take on various shapes, including linear, cyclic (both aromatic and aliphatic), and cage-like. Compare to linear molecules, cyclic and cage-like molecules contain less rotational bonds and show lower conformational flexibility. In particular, existence of sp^2 hybridized atoms in aromatic rings leads to the extremely rigid ring structure, atoms in which can barely move (Supplementary Fig. 19). This structural complexity, in other words, the cyclicity of molecules can be described by employing the kappa index ^3k. The definition of ^3k based on the count of three-bond fragment ^3P in molecular graph and the number of atoms A. In addition, size contribution of different atoms is also considered and evaluated by the ratio of covalent radii between atom i and C(sp^3), which is defined as $\alpha_\mathrm{i} = (r_i/r_{\mathrm{CSP3}}) - 1$ (Supplementary Table 8). The value of ^3k decreases as the degree of cyclicity of molecular skeleton increases (Supplementary Fig. 20). The second case is the flexibility of the alkyl tail, which is related to the tail curling of organic spacers in the perovskite lattice. The geometry of alkyl tail of organic spacers highly influences the hydrogen bonds between organic spacers and inorganic layers in 2D perovskite, directly leading to the distortion and tilting of inorganic octahedra or instability of 2D perovskites. For example, the structure of 2D perovskites with different phenylalkylammonium cations exhibits different connection modes of inorganic layers.[7] Therefore, the number of rotational bonds in the alkyl tail $\mathrm{Num_{Rot}}$ is considered a feature to assess the flexibility of the alkyl tail.

Support vector classification (SVC) algorithm. Machine learning techniques have been extensively utilized in the material science. Since the training dataset used in this work is small, the SVC algorithm with linear kernel is applied, which showed great

performance on the small-scale dataset.[8] For binary classification task, the SVC find the smallest distance between decision boundary and samples, to accurately classify positive and negative samples with the smallest generalization error. Training dataset contains N input individuals x_1, x_2, …, x_N, with target property t_1, t_2, …, t_N, where $t_n \in \{-1. 1\}$. The SVC model with linear kernel is: $y(\mathrm{x}) = \mathrm{w}^{\mathrm{T}}\varphi(\mathrm{x}) + \mathrm{b}$. Where $\varphi(\mathrm{x})$ represents fixed feature-space transformation, and w and b represent the normal vector to the hyperplane and bias parameter, respectively. The margin between sample x_n and hyperplane defined by $y(\mathrm{x})$ is $\frac{t_n y(\mathrm{x})}{||\mathrm{w}||}$, and the solution of maximum margin is found by solving:

$$arg \max_{w,b} \left\{ \frac{1}{||w||} \min_{n} [t_n(\mathrm{w}^{\mathrm{T}}\varphi(\mathrm{x}) + \mathrm{b})] \right\} \quad (1)$$

On the basis of coefficients obtained from the training process of SVC model, the target property can be predicted as follows:

$$\mathrm{P} = -1.98 \times \mathrm{Dis_{NN}} - 2.24 \times \mathrm{STEI} - 1.04 \times \mathrm{Eccentricity} - 1.58 \times \mathrm{Num_N} \\ +2.16 \times \mathrm{Num_{Rot}} - 0.03 \times \mathrm{MolWt} + 14.01 \quad (2)$$

Cross-validation. Cross-validation is a popular model validation technique to assess the generalization ability of ML models on the dataset of unknown data. Different portions of dataset are utilized to train and test the model on different iterations. The dataset is split into k groups in the procedure of k-fold cross-validation, and each instance is assigned to an individual group and stays in that group for the duration of the procedure. The ML model is trained by using training set with k-1 of the folds, and the trained model is validated on the remaining part of data. Each instance in the dataset should be used to train the model k-1 times and to valide the model 1 time. In this work, the 10-fold cross-validation is utlized to train SVC model for 2D AgBi iodide perovskites.

Model evaluation for SVC model. Model evaluation citerations are crucial for assessing the performation of ML models. In this work, area under curve (AUC) is utlized to evluate the accuracy of SVC model, and confusion matrix is applied to

measure the count of correct and incorrect predictions obtained from classifier. The classification models produce the prediction probability for samples. The classification threshold is set to 0.5 in this work, and the prediction probability results of samples are compared to the pre-defined threshold. The prediction probability results correspond to the probabilities that samples belong to positive class (i.e. 2D perovskites) or negative class (i.e. non-2D perovskites). Therefore, samples with prediction probability results larger than 0.5 are classified into positive class, and samples with prediction probability results less than 0.5 are classified into negative class.

According to the classification results, the count of positive samples predicted correctly is defined as true positive (TP), and the count of positive samples predicted falsely is defined as false positive (FP). The count of negative samples predicted correctly is defined as true negative (TN), and the count of negative samples predicted falsely is defined as false negative (FN). By calculating the values of TP, FP, TN and FN, we obtained the confusion matrix, which represents the counts of the predicted classes versus the true classes of test set. The confusion matrix is shown as follows:

	Predicted positive	Predicted negative
True positive	TP	FN
True negative	FP	TN

The true positive rate (TPR) and false positive rate (FPR) are calculated based on TP, TN, FP and FN.

$$\mathrm{TPR} = \frac{\mathrm{TP}}{\mathrm{TP+FN}} \tag{3}$$

$$\mathrm{FPR} = \frac{\mathrm{FP}}{\mathrm{TN+FP}} \tag{4}$$

The receiver operating characteristic (ROC) curve can be drawn using TPR and FPR as coordinates, and is often used to measure the performance of classification models. When comparing the performance of different classification models, if the ROC curve of one model is completely below the ROC curve of the other, it means that the performance of the latter model is better than the former. If the ROC curves of two classification models intersect, the comparison is difficult. Thus the more appropriate model evaluation index is the AUC value. The higher AUC value corresponds to the

better performance of classification model. The AUC value of a classification model without learning algorithm is equal to 0.5, and the AUC value of the perfect classification model is equal to 1.

SHapley Additive exPlanations (SHAP) analysis. SHAP is developed for explaination individual predictions by Lundberg and Lee (2017), which is based on the game theoretically optimal Shapley values.[9] As an model-agnostic interpretation approach, SHAP analysis is utilized to explain the prediction of a given instance by calculating the contribution of features to the final prediction. For SVC model of synthesis feasibility of 2D AgBi iodide perovskites, SHAP values of six features are computed to measure the contribution of each feature. The SHAP values can be obtained as follows:

$$\Phi_i = \sum_{S \subseteq F,\{i\}} \frac{|S|!(|F|-|S|-1)!}{|F|!} \left[f_{S\cup\{i\}}\left(x_{S\cup\{i\}}\right) - f_s(x_s)\right] \quad (5)$$

Here, Φ_i is the SHAP value of feature i, the feature set S is the subset of the whole feature set F. x_S and f_S represent the values of the input features in the feature set S and the trained model with the feature set S, respectively. By calculating the change of model output when the feature i is added to the feature set S, the contribution of the feature i to the model output can be quantitative determined.

T-distributed Stochastic Neighbourhood Embedding (t-SNE) algorithm. T-SNE is an unsupervised ML techniques, which is used to visualise the high-dimensional data in two- or three-dimensions.[10] T-SNE algorithm applies the heavy-tailed student t-distribution to calculate the similarity between two data in the high-dimensional space. To visualize dataset of synthesis feasibility of 2D AgBi iodide perovskites, the data with 24 generally descriptors obtained from RDKit is utilized as the input distribution, and t-SNE algorithm is utilized to reduce the 24-dimensional data to two-dimensional data. For a dataset contains N high-dimensional sample x_1, x_2, …, x_N, the similarity between x_i and x_j is calculated as follows:

$$q_{ij} = \frac{(1+||x_i - x_j||^2)^{-1}}{\sum_k \sum_{l \neq k} (1+||x_k - x_l||^2)^{-1}} \quad (6)$$

Where $q_{ii} = 0$.

The location of points in the map are obtained by minimizing the (non-symmetric) Kullback-Leibler divergence between the input distribution P and the embedding distribution Q, as follows:

$$\mathrm{KL}(P||Q) = \sum_{i \neq j} p_{ij} log \frac{p_{ij}}{q_{ij}} \quad (7)$$

The results of this optimization reflect the simlarities between data in high-dimensional space.

Density functional theory calculations.

The electronic structure of eight synthesized perovskites were calculated by utilizing the Vienna Ab initio Simulation Package 5.4 (VASP).[11] The projector-augmented wave (PAW) method[12] with the generalized gradient approximation of Perdew–Burke–Ernzerhof (PBE) exchange-correlation functional[13] was adopted. The Heyd–Scuseria–Ernzerhof (HSE06) hybrid functional[14,15] was applied to compute the electronic structure more accurately. The van der Waals interaction was taken account into by using Grimme's D3 correlation.[16] The cut-off energy was set as 520 eV for structural optimization, and 400 eV for electronic structure calculations. The convergence thresholds of force and energy difference were set as 2×10^{-2} eV Å^{-1} and 1×10^{-4} eV, respectively.

Supplementary Notes

Note 1. To compare the inorganic frameworks of 2D Pb perovskites and AgBi perovskites, the metal-iodide bond length, metal-metal bond length, and penetration depth of single-layer $(C_4NH_{12})_2PbI_4$ and $(C_4NH_{12})_4AgBiI_8$ were calculated (Supplementary Fig. 14 and Supplementary Table 7). The metal-iodide bond length and metal-metal bond length of 2D perovskites are measured in the Vesta software[17]. Note that the hydrogen bond formed by the protonated nitrogen atom of organic spacers and axial halogen atoms of inorganic layer has an influence on the distortion of octahedra.

The penetration depth of organic spacers is defined as the average distance between the protonated nitrogen atoms of organic spacers and the terminal halogen of inorganic layers.[18] Moreover, to assess the softness of inorganic framework of Pb perovskites and AgBi perovskites, the Young's modulus of $(CH_3NH_3)_2AgBiI_6$ and $CH_3NH_3PbI_3$ were calculated.

Note 2. To visualise prediction results of all compounds in prediction set, a Python program named visualize.ipynb is provided. Running this program requires the t-SNE results of dataset, *i.e.*, the file predict_result.xlsx. With these given files, run visualize.ipynb to visualize the results of prediction set, and click the point in the map to output all compounds near that point. The output figure is named by ID_predicted P value. Notably, the predicted P value is set as -50 for compounds out of the region obtained by subgroup discovery.

Note 3. The synthesis feasibility of potential organic spacers for 2D AgBi perovskites are classified into three categories based on their P values (Supplementary Fig. 22-24), namely, superior synthesis feasibility (larger than 4), high synthesis feasibility (from 2 to 4), and moderate synthesis feasibility (from 0 to 2).

Supplementary Figures

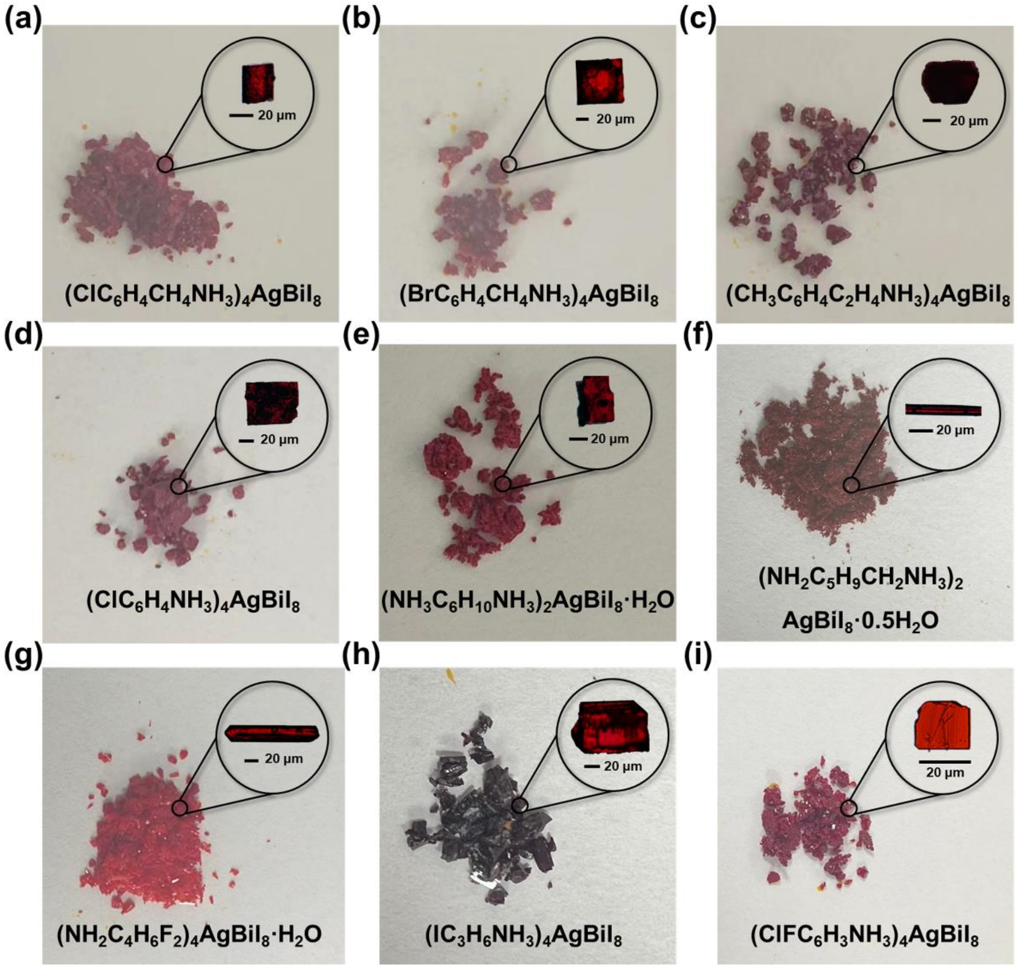

Supplementary Figure 1. Optical images of synthesized perovskites. Optical image of (a) $(ClC_6H_4CH_4NH_3)_4AgBiI_8$, (b) $(BrC_6H_4CH_4NH_3)_4AgBiI_8$, (c) $(CH_3C_6H_4C_2H_4NH_3)_4AgBiI_8$, (d) $(ClC_6H_4NH_3)_4AgBiI_8$, (e) $(NH_3C_6H_{10}NH_3)_2AgBiI_8 \bullet H_2O$, (f) $(NH_2C_5H_9CH_2NH_3)_2AgBiI_8 \bullet 0.5H_2O$, (g) $(NH_2C_4H_6F_2)_4AgBiI_8 \bullet H_2O$, (h) $(IC_3H_6NH_3)_4AgBiI_8$, and (i) $(ClFC_6H_3NH_3)_4AgBiI_8$.

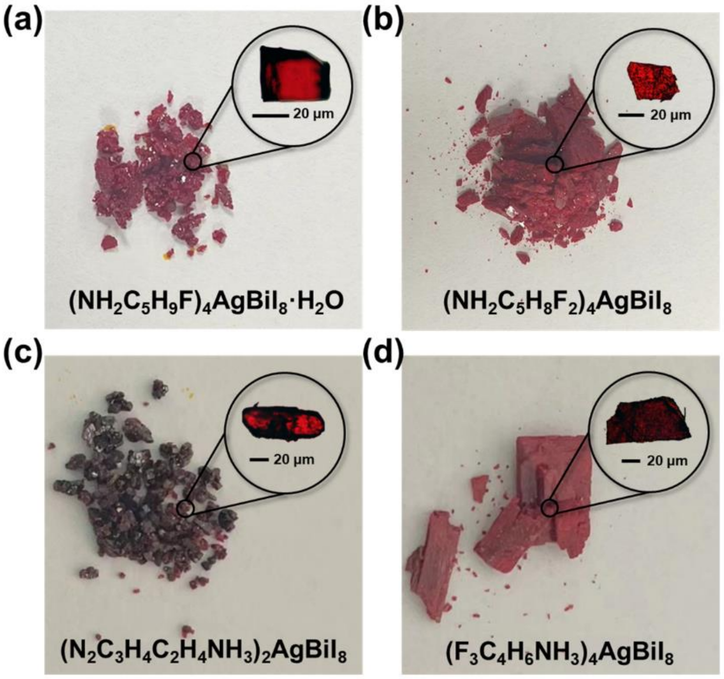

Supplementary Figure 2. Optical images of synthesized perovskites. Optical image of (a) $(NH_2C_5H_9F)_4AgBiI_8 \cdot H_2O$, (b) $(NH_2C_5H_8F_2)_4AgBiI_8$, (c) $(N_2C_3H_4C_2H_4NH_3)_2AgBiI_8$, and (d) $(F_3C_4H_6NH_3)_4AgBiI_8$.

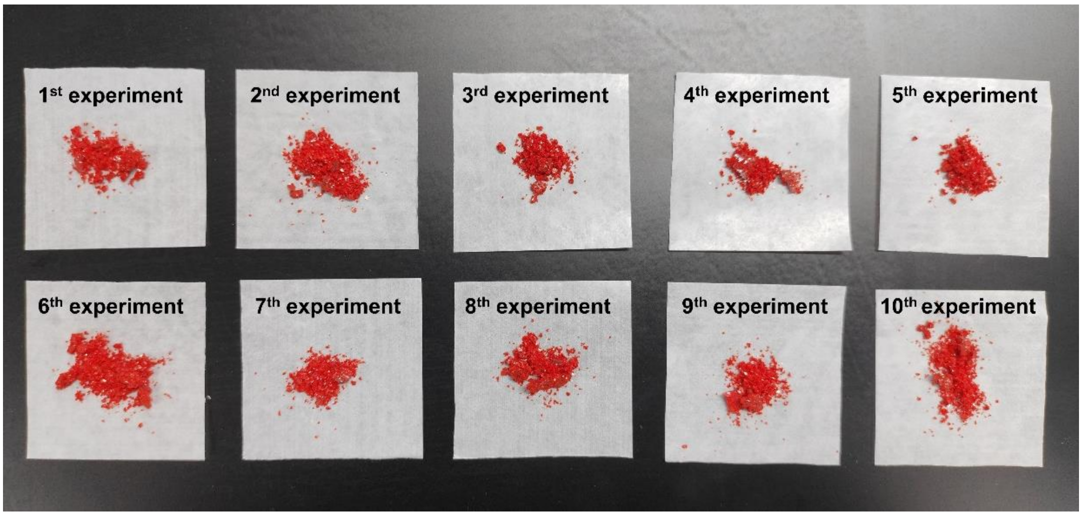

Supplementary Figure 3. Images of $(NH_2C_5H_8F_2)_4AgBiI_8$ synthesized in ten synthesis experiments.

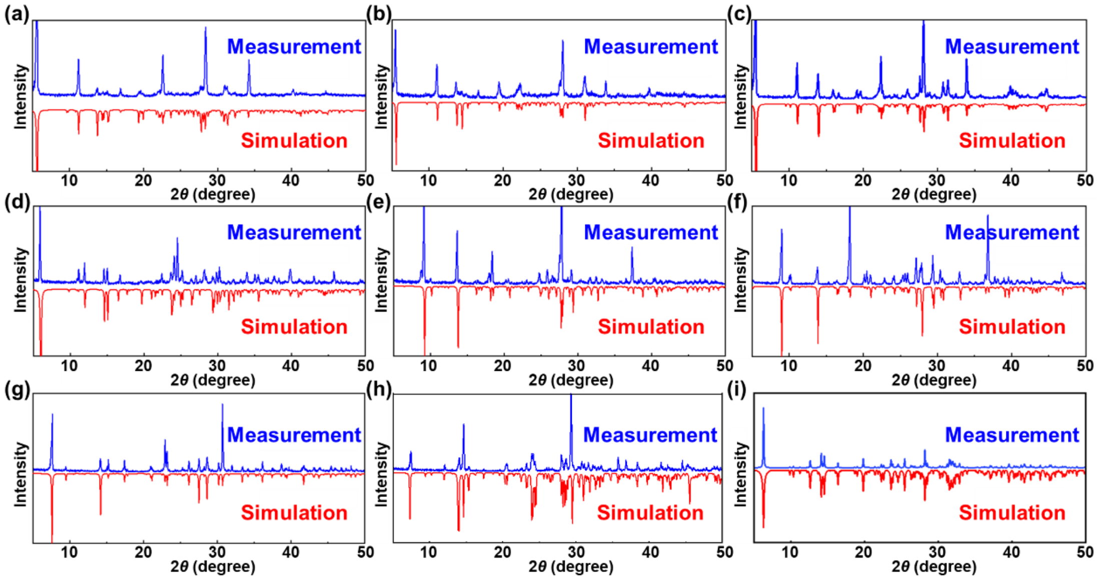

Supplementary Figure 4. Patterns of the powder X-ray diffraction of synthesized perovskites. Patterns of the powder X-ray diffraction of (a) $(ClC_6H_4CH_4NH_3)_4AgBiI_8$, (b) $(BrC_6H_4CH_4NH_3)_4AgBiI_8$, (c) $(CH_3C_6H_4C_2H_4NH_3)_4AgBiI_8$, (d) $(ClC_6H_4NH_3)_4AgBiI_8$, (e) $(NH_3C_6H_{10}NH_3)_2AgBiI_8$•$H_2O$, (f) $(NH_2C_5H_9CH_2NH_3)_2AgBiI_8$•$0.5H_2O$, (g) $(NH_2C_4H_6F_2)_4AgBiI_8$•H_2O, (h) $(IC_3H_6NH_3)_4AgBiI_8$, and (i) $(ClFC_6H_3NH_3)_4AgBiI_8$.

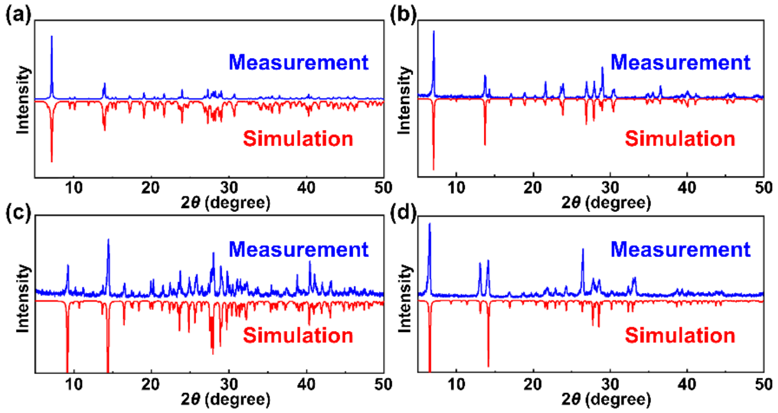

Supplementary Figure 5. Patterns of the powder X-ray diffraction of synthesized perovskites. Patterns of the powder X-ray diffraction of (a) $(NH_2C_5H_9F)_4AgBiI_8$•H_2O, (b) $(NH_2C_5H_8F_2)_4AgBiI_8$, (c) $(N_2C_3H_4C_2H_4NH_3)_2AgBiI_8$, and (d) $(F_3C_4H_6NH_3)_4AgBiI_8$.

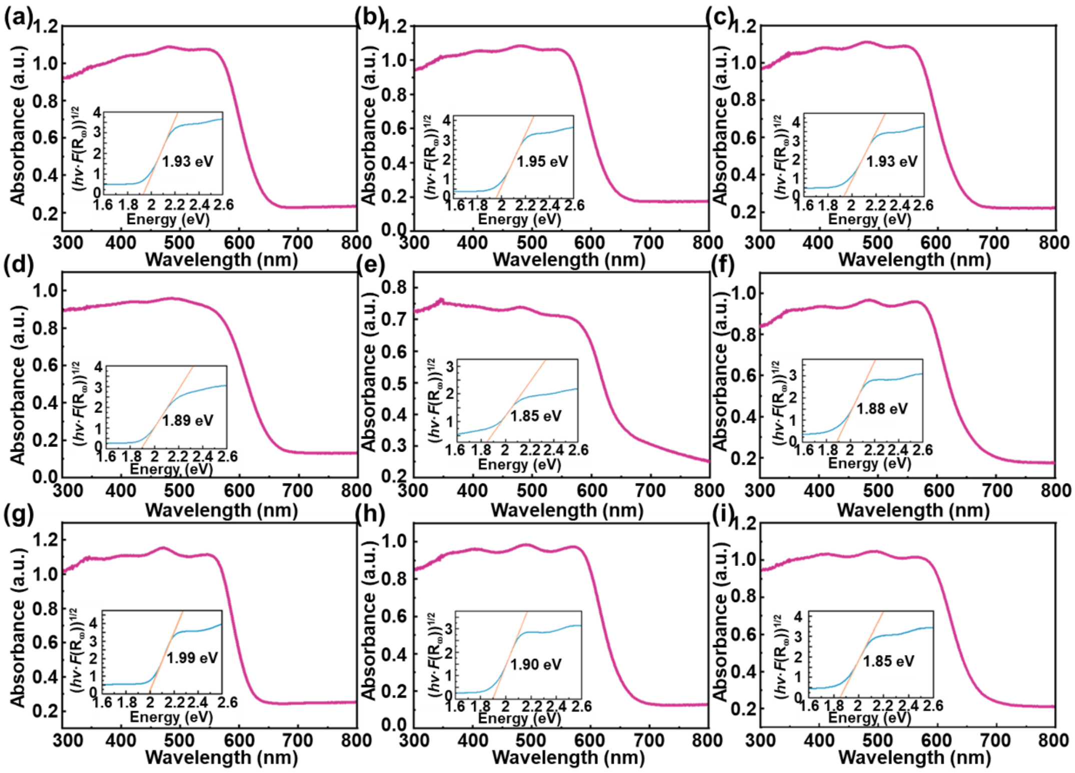

Supplementary Figure 6. UV-vis absorption spectra of synthesized perovskites. UV-vis absorption spectra of (a) $(ClC_6H_4CH_4NH_3)_4AgBiI_8$, (b) $(BrC_6H_4CH_4NH_3)_4AgBiI_8$, (c) $(CH_3C_6H_4C_2H_4NH_3)_4AgBiI_8$, (d) $(ClC_6H_4NH_3)_4AgBiI_8$, (e) $(NH_3C_6H_{10}NH_3)_2AgBiI_8•H_2O$, (f) $(NH_2C_5H_9CH_2NH_3)_2AgBiI_8•0.5H_2O$, (g) $(NH_2C_4H_6F_2)_4AgBiI_8•H_2O$, (h) $(IC_3H_6NH_3)_4AgBiI_8$, and (i) $(ClFC_6H_3NH_3)_4AgBiI_8$.

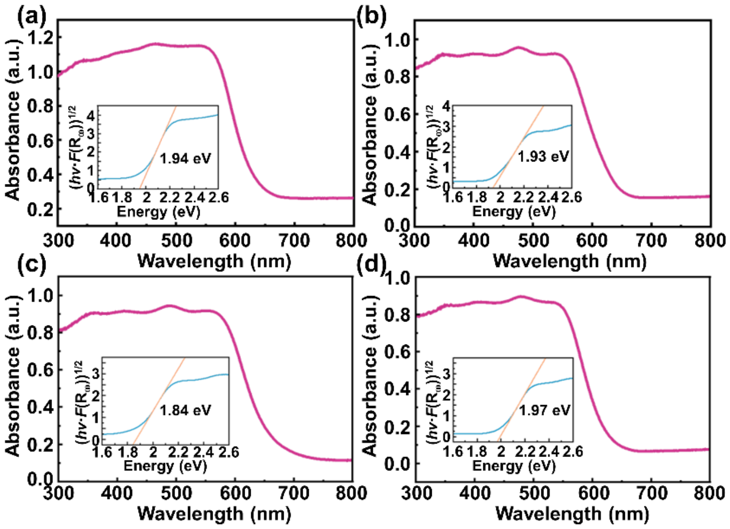

Supplementary Figure 7. UV-vis absorption spectra of synthesized perovskites. UV-vis absorption spectra of (a) $(NH_2C_5H_9F)_4AgBiI_8•H_2O$, (b) $(NH_2C_5H_8F_2)_4AgBiI_8$, (c)

$(N_2C_3H_4C_2H_4NH_3)_2AgBiI_8$, and (d) $(F_3C_4H_6NH_3)_4AgBiI_8$.

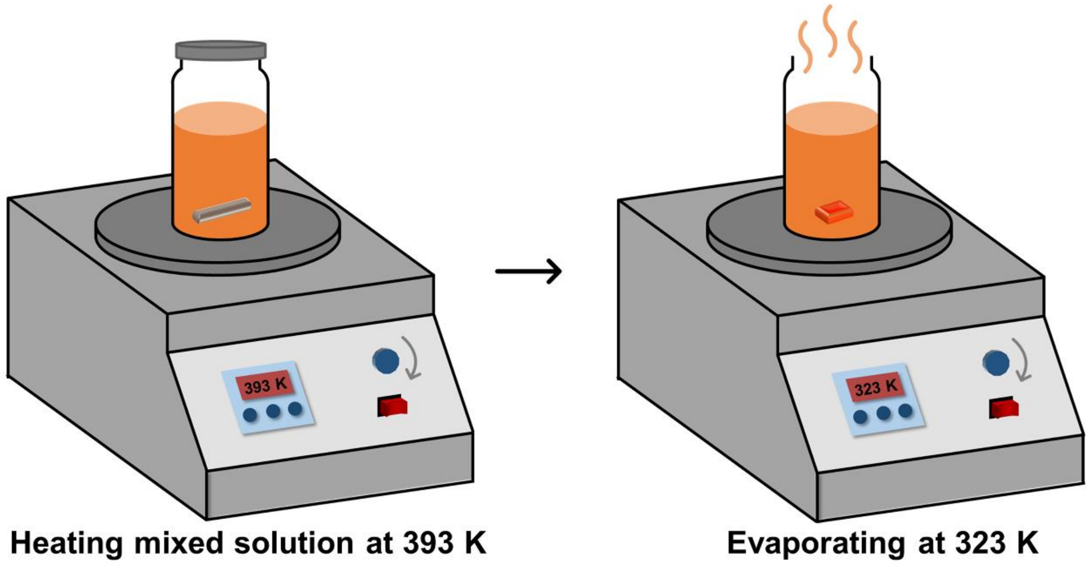

Supplementary Figure 8. Schematic sketch of experimental synthesis method.

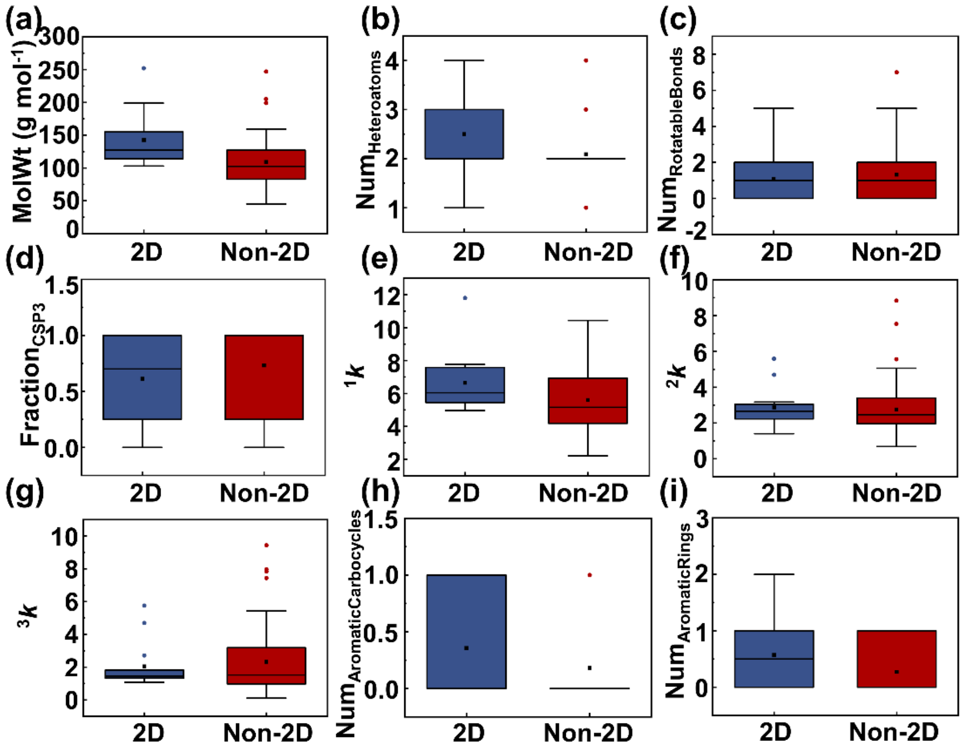

Supplementary Figure 9. Visualizing synthesis feasibility of 80 compounds with material descriptors. Box plot of synthesis feasibility of 80 compounds with varied (a) MolWt, (b) $Num_{Heteroatoms}$, (c) $Num_{RotatableBonds}$, (d) $Fraction_{CSP3}$, (e) $^1\kappa$, (f) $^2\kappa$, (g) $^3\kappa$, (h) $Num_{AromaticCarbocycles}$, and (i) $Num_{AromaticRings}$. In each boxplot, the central thick black line

represents the median, color shaded boxes represent the first and third quartiles (the 25th and 75th percentiles), and the whiskers extend no further than 1.5 times of the distance between the first and third quartiles.

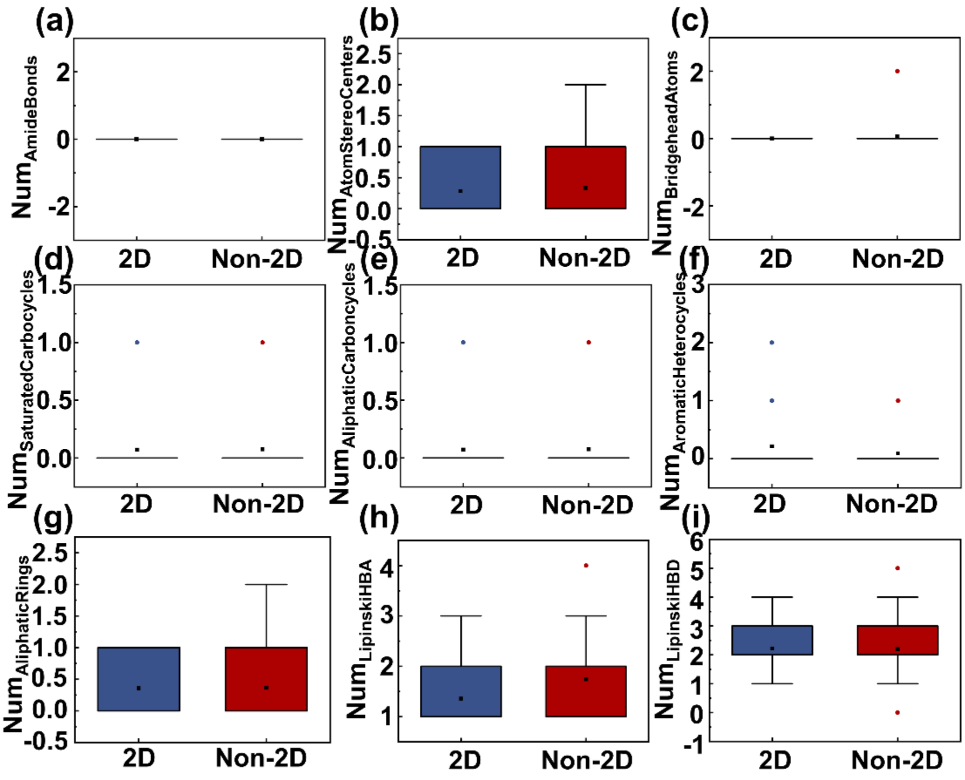

Supplementary Figure 10. Visualizing synthesis feasibility of 80 compounds with material descriptors. Box plot of synthesis feasibility of 80 compounds with varied (a) $Num_{AmideBonds}$, (b) $Num_{AtomStereoCenters}$, (c) $Num_{BridgeheadAtoms}$, (d) $Num_{SaturatedCarbocycles}$, (e) $Num_{AliphaticCarbocycles}$, (f) $Num_{AromaticHeterocycles}$, (g) $Num_{AliphaticRings}$, (h) $Num_{LipinskiHBA}$, and (i) $Num_{LipinskiHBD}$. In each boxplot, the central thick black line represents the median, color shaded boxes represent the first and third quartiles (the 25th and 75th percentiles), and the whiskers extend no further than 1.5 times of the distance between the first and third quartiles.

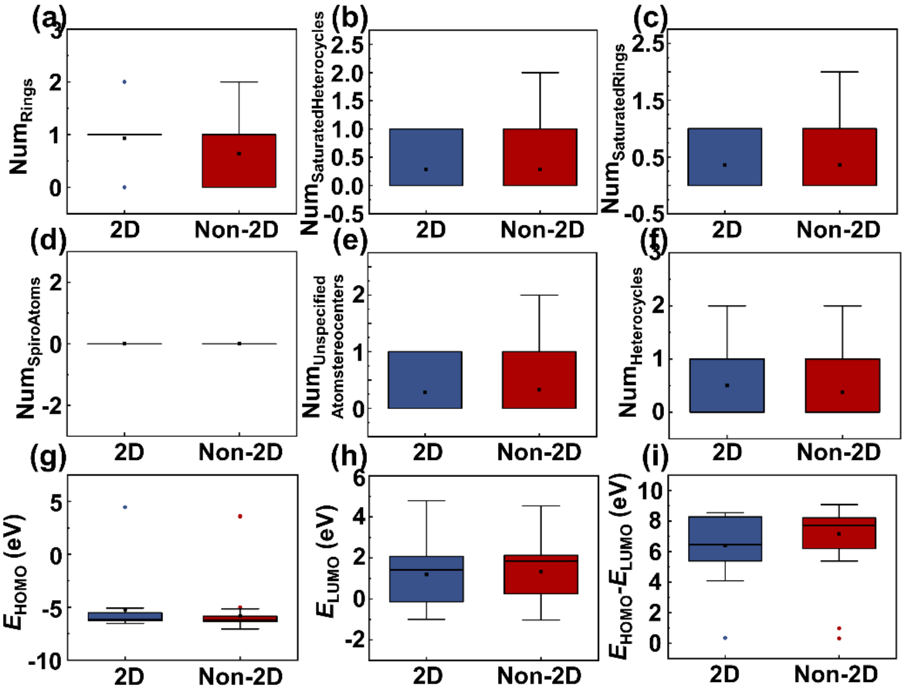

Supplementary Figure 11. Visualizing synthesis feasibility of 80 compounds with material descriptors. Box plot of synthesis feasibility of 80 compounds with varied (a) Num_{Rings}, (b) $Num_{SaturatedHeterocycles}$, (c) $Num_{SaturatedRings}$, (d) $Num_{SpiroAtoms}$, (e) $Num_{UnspecifiedAtomStereoCenters}$, (f) $Num_{Heterocycles}$, (g) E_{HOMO}, (h) E_{LUMO}, and (i) E_{HOMO}-E_{LUMO}. In each boxplot, the central thick black line represents the median, color shaded boxes represent the first and third quartiles (the 25th and 75th percentiles), and the whiskers extend no further than 1.5 times of the distance between the first and third quartiles.

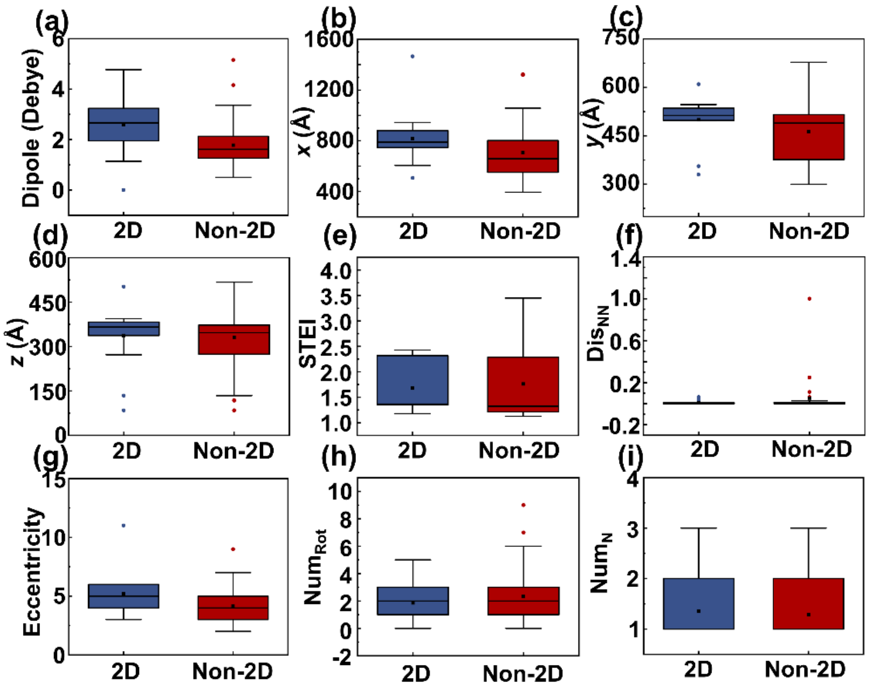

Supplementary Figure 12. Visualizing synthesis feasibility of 80 compounds with material descriptors. Box plot of synthesis feasibility of 80 compounds with varied (a) Dipole, (b) x, (c) y, (d) z, (e) STEI, (f) Dis_{NN}, (g) Eccentricity, (h) Num_{Rot}, and (i) Num_{N}. In each boxplot, the central thick black line represents the median, color shaded boxes represent the first and third quartiles (the 25th and 75th percentiles), and the whiskers extend no further than 1.5 times of the distance between the first and third quartiles.

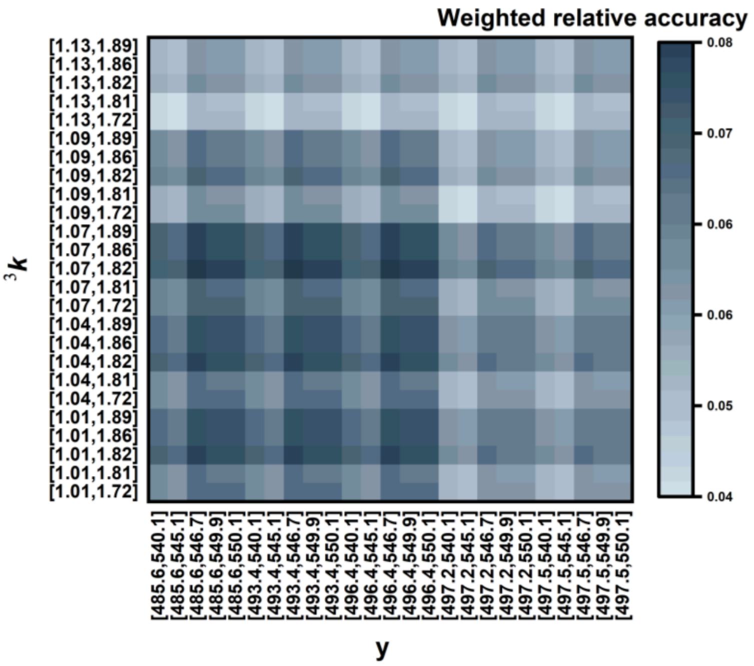

Supplementary Figure 13. Weighted relative accuracy of all subgroups with y ranging

from 486 pm to 550 pm and ^{3}k ranging from 1.01 to 1.89, respectively.

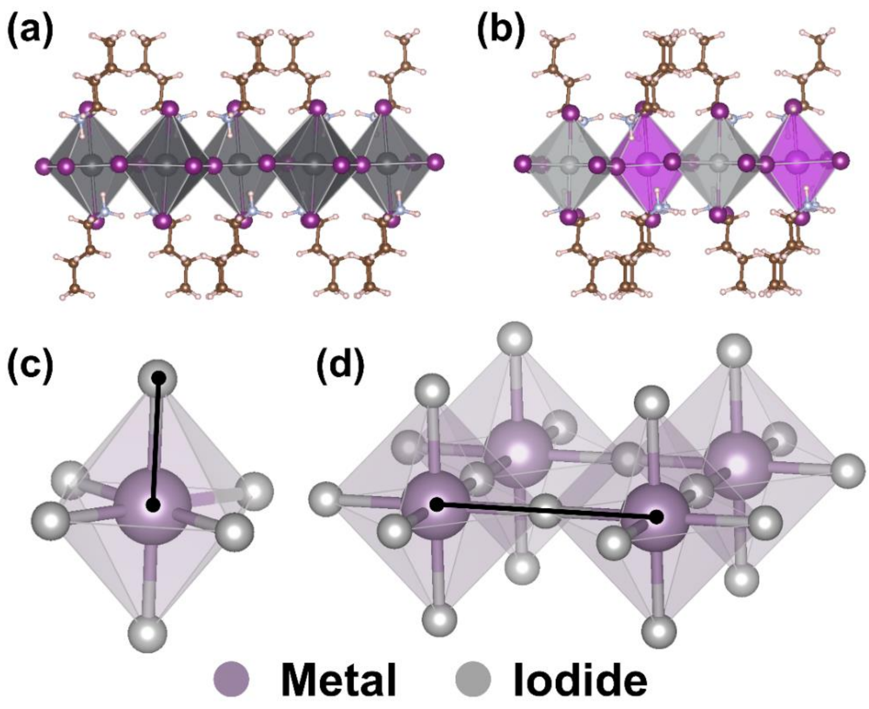

Supplementary Figure 14. Schematic diagram of 2D perovskite structure. Crystal structure of (a) $(C_4NH_{12})_2PbI_4$ and (b) $(C_4NH_{12})_4AgBiI_8$. The schematic diagram of (c) metal-iodide bond length and (d) metal-metal bond length.

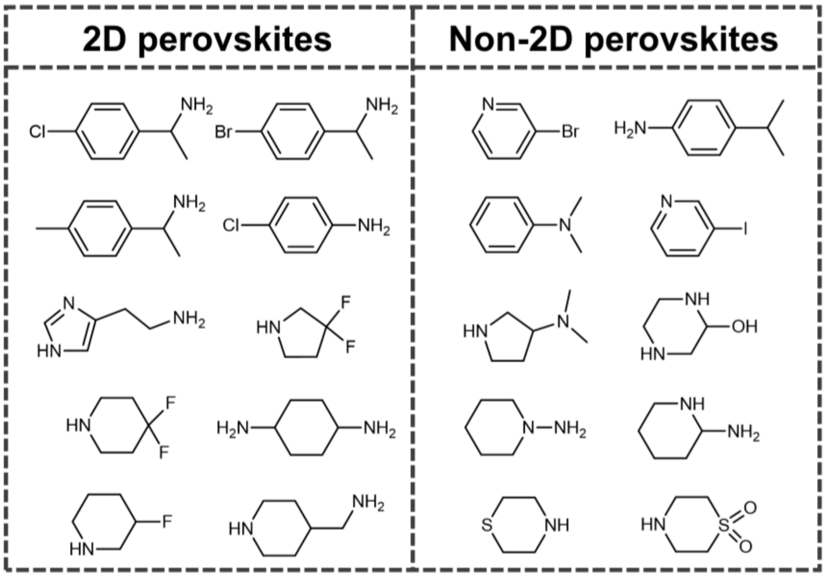

Supplementary Figure 15. Organic spacers in the statically "most interesting" subgroup, which contain 10 organic spacers of 2D AgBi iodide perovskites and 10

organic spacers of non-2D AgBi iodide perovskites.

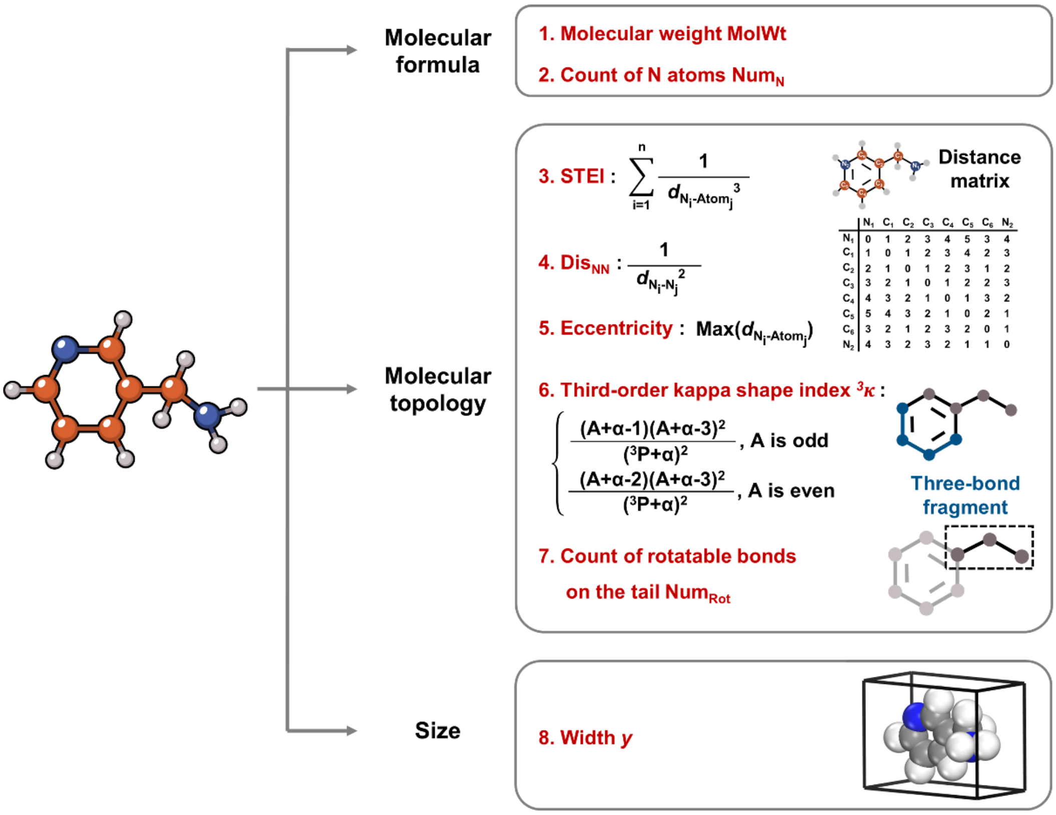

Supplementary Figure 16. Schematic sketch of the molecular representation. Three aspects of molecular features are considered, namely, molecular formula, molecular topology, and size. Here, $d_{\mathrm{N_i-Atom_j}}$ represents the topological distance between the nitrogen i and atom j in the molecular skeleton, $d_{\mathrm{N_i-N_j}}$ represents the topological distance between nitrogen i and j. A and ^{3}P represent the count of atoms in the molecular skeleton and three-bond fragments.

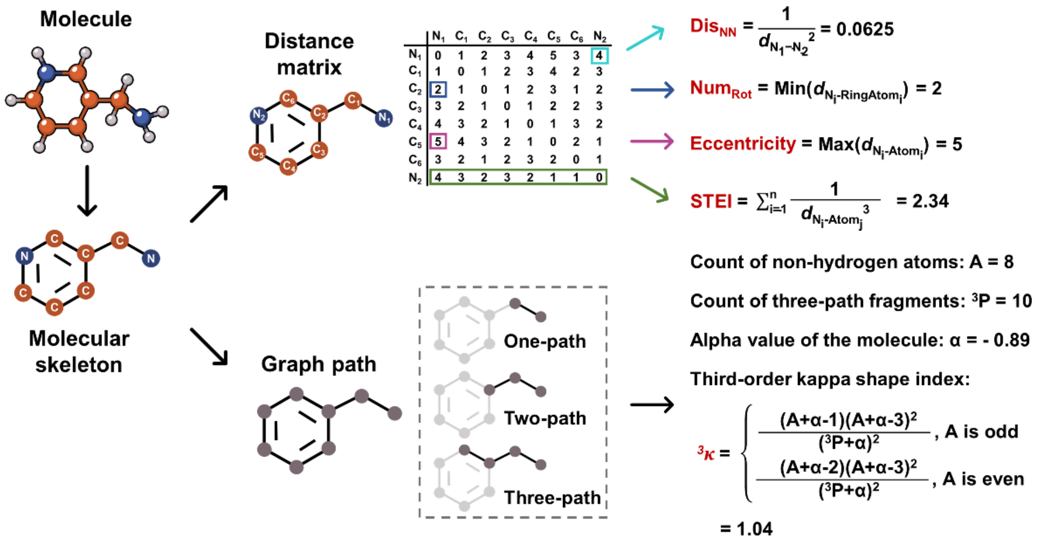

Supplementary Figure 17. Schematic sketch of the molecular topology. Here, $d_{\mathrm{N_i-Atom_j}}$ represents the topological distance between the nitrogen i and atom j in the molecular skeleton, $d_{\mathrm{N_i-N_j}}$ represents the topological distance between nitrogen i and j, $d_{\mathrm{N_i-Atom_j}}$ represents the topological distance between the nitrogen i and atom j in the ring.

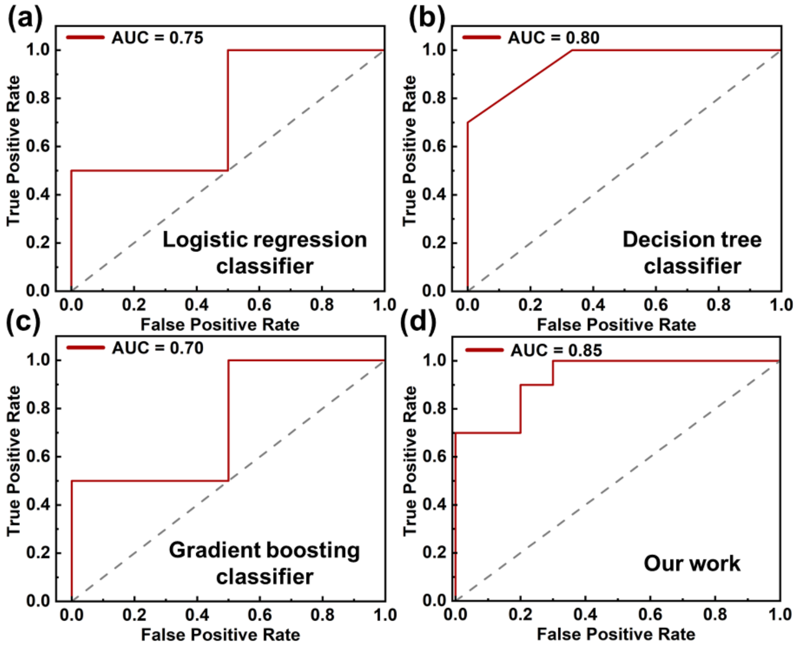

Supplementary Figure 18. Model comparison results. Model performance of (a) logistic regression classifier, (b) decision tree classifier, (c) gradient boosting classifier, and (d) support vector classifier in our work.

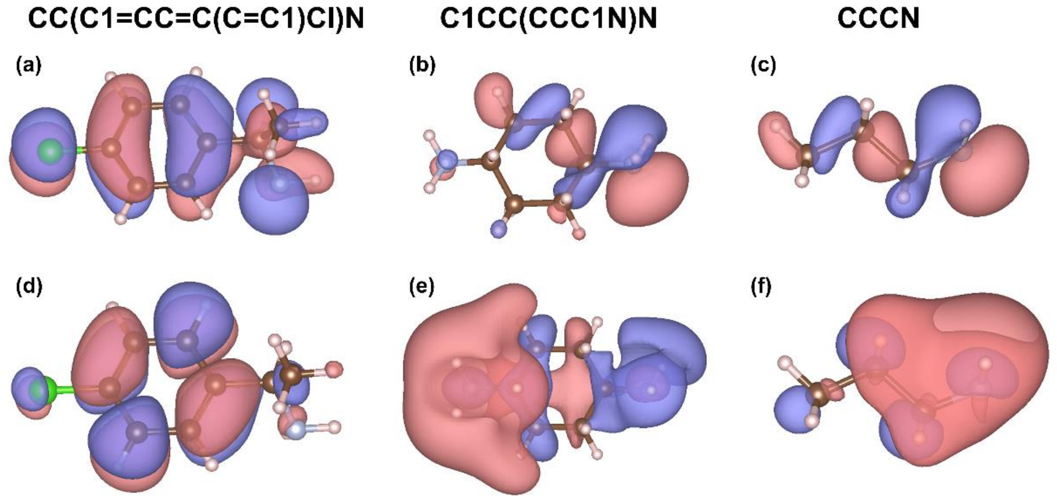

Supplementary Figure 19. Molecular frontier orbitals. HOMO orbital plot of (a) CC(C1=CC=C(C=C1)Cl)N, (b) C1CC(CCC1N)N, and (c) CCCN. The LUMO orbital plot of (d) CC(C1=CC=C(C=C1)Cl)N, (e) C1CC(CCC1N)N, and (f) CCCN.

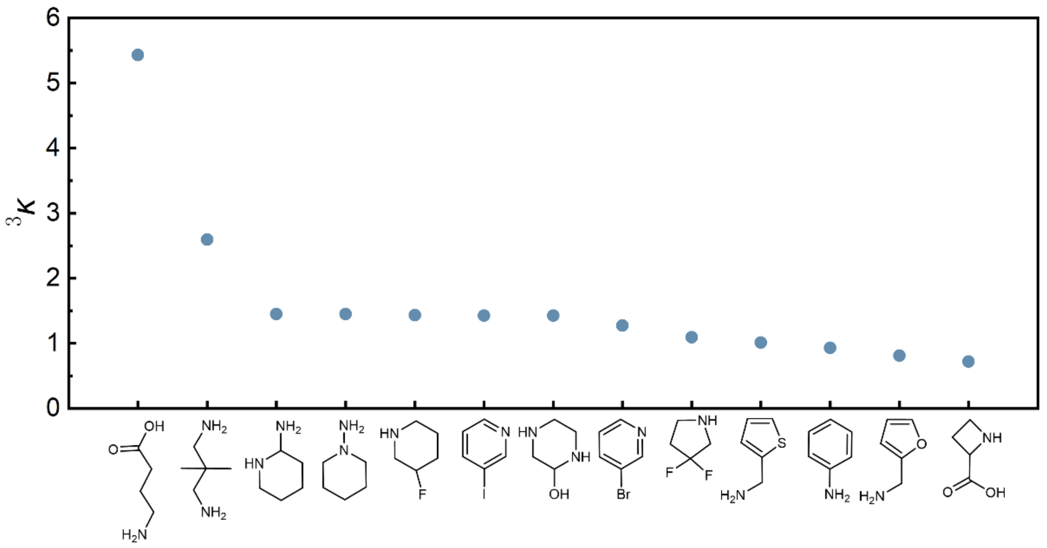

Supplementary Figure 20. Third-ordered kappa index ^{3}k of different organic spacers.

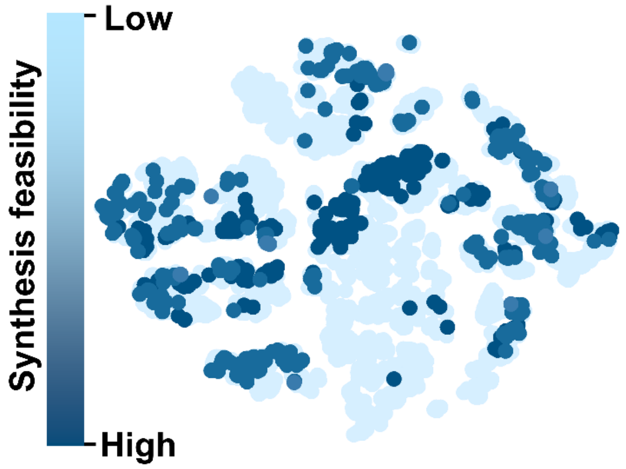

Supplementary Figure 21. Predicted synthesis feasibility of prediction set visualized by t-SNE mothod.

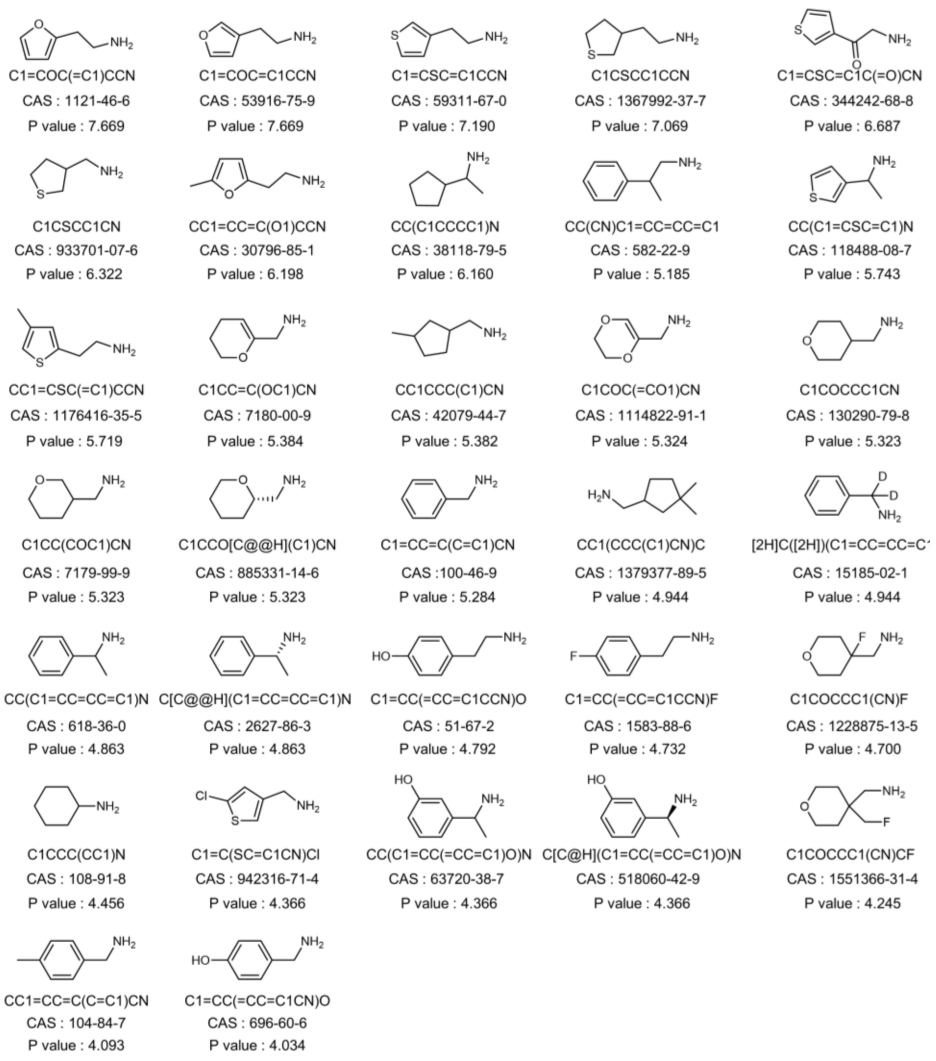

Supplementary Figure 22. Molecular graph of commercially available candidates

with superior synthesis feasibility.

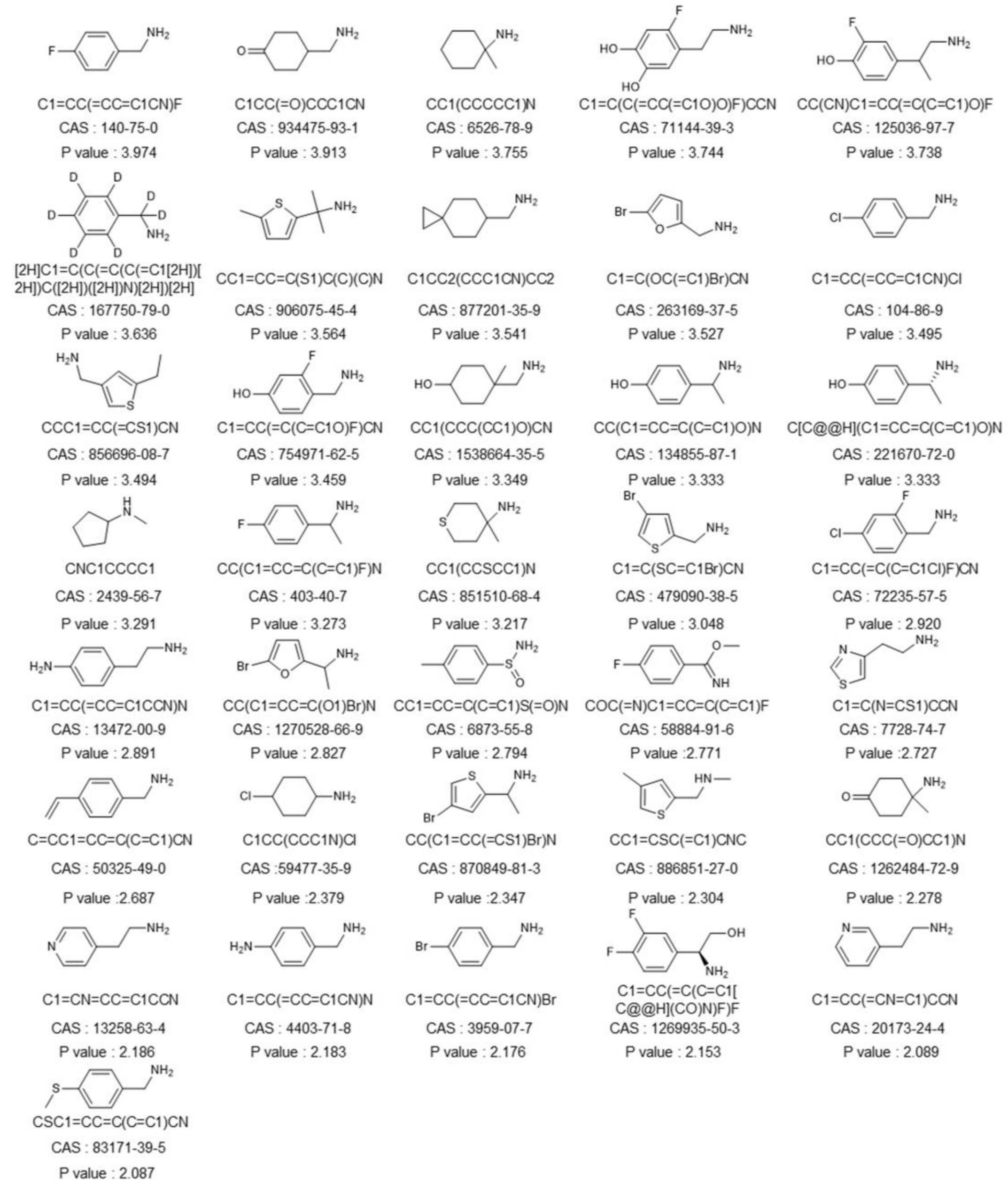

Supplementary Figure 23. Molecular graph of commercially available candidates with high synthesis feasibility.

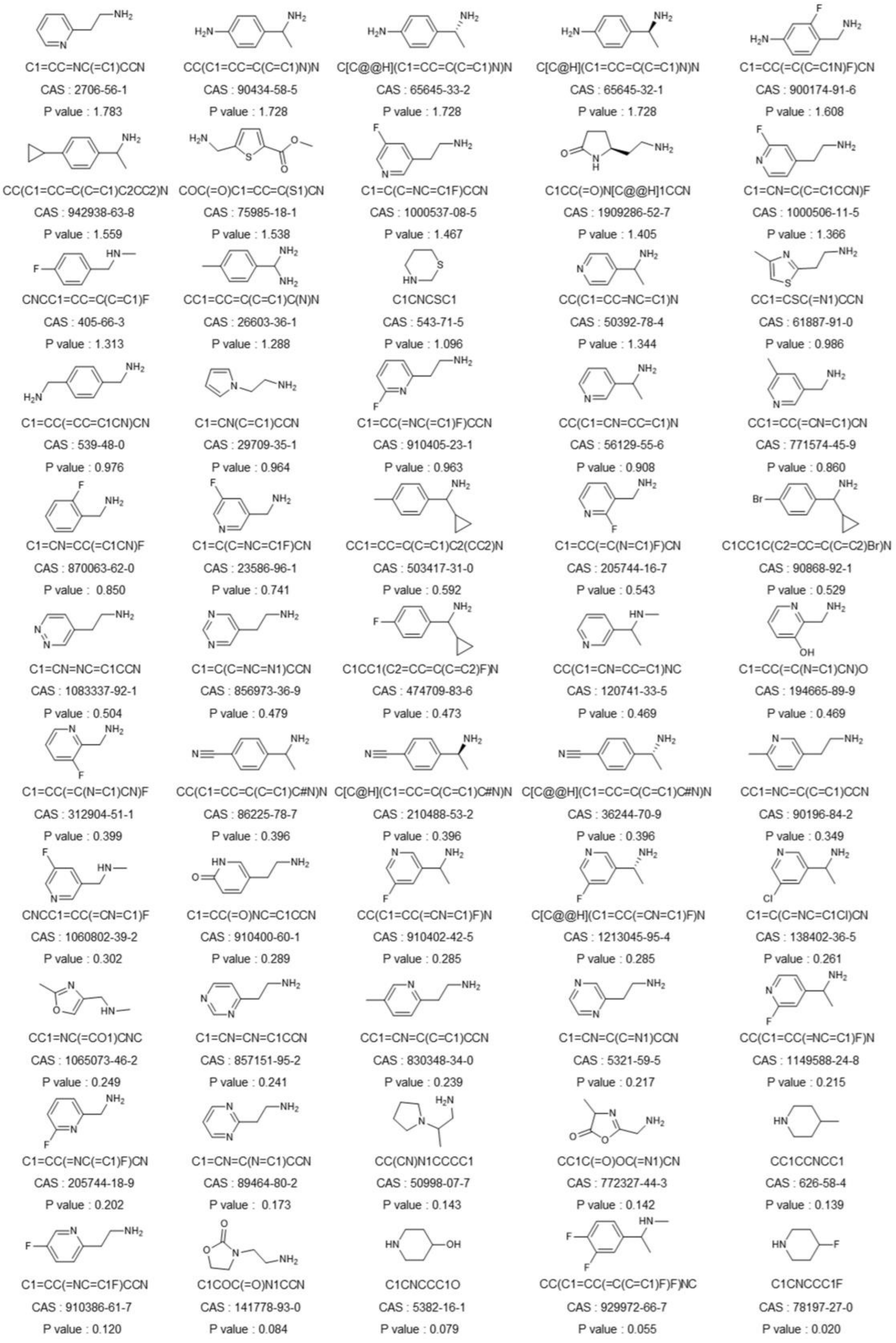

Supplementary Figure 24. Molecular graph of commercially available candidates with moderate synthesis feasibility.

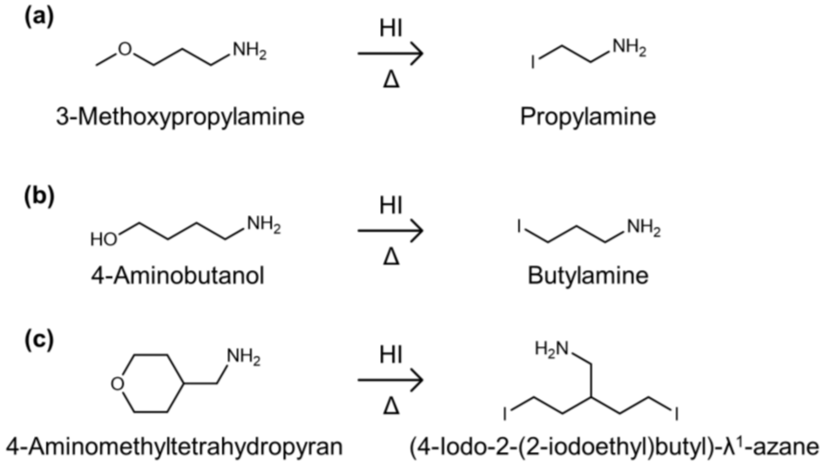

Supplementary Figure 25. Reactions between amines and HI solution. The reaction of (a) 3-Methoxypropylamine, (b) 4-Aminobutanol,[19] and (c) 4-Aminomethyltetrahydropyran in HI solution.

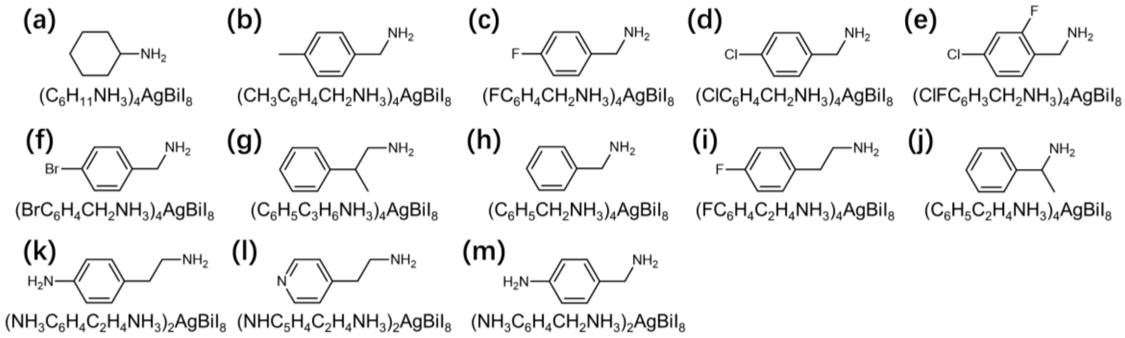

Supplementary Figure 26. Organic spacers of 2D AgBi iodide perovskites in the validation set. Organic spacers of (a) $(C_6H_{11}NH_3)_4AgBiI_8$, (b) $(CH_3C_6H_4CH_2NH_3)_4AgBiI_8$, (c) $(FC_6H_4CH_2NH_3)_4AgBiI_8$, (d) $(ClC_6H_4CH_2NH_3)_4AgBiI_8$, (e) $(ClFC_6H_3CH_2NH_3)_4AgBiI_8$, (f) $(BrC_6H_4CH_2NH_3)_4AgBiI_8$, (g) $(C_6H_5C_3H_6NH_3)_4AgBiI_8$, (h) $(C_6H_5CH_2NH_3)_4AgBiI_8$, (i) $(FC_6H_4C_2H_4NH_3)_4AgBiI_8$, (j) $(C_6H_5C_2H_4NH_3)_4AgBiI_8$, (k) $(NH_3C_6H_4C_2H_4NH_3)_2AgBiI_8$, (l) $(NHC_5H_4C_2H_4NH_3)_2AgBiI_8$, and (m) $(NH_3C_6H_4CH_2NH_3)_2AgBiI_8$.

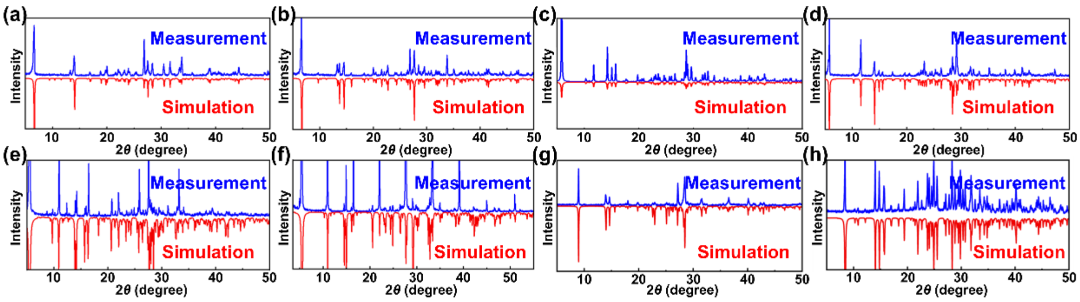

Supplementary Figure 27. Patterns of the powder X-ray diffraction of synthesized perovskites. Patterns of the powder X-ray diffraction of (a) $(C_6H_{11}NH_3)_4AgBiI_8$, (b) $(FC_6H_4CH_2NH_3)_4AgBiI_8$, (c) $(ClC_6H_4CH_2NH_3)_4AgBiI_8$, (d) $(BrC_6H_4CH_2NH_3)_4AgBiI_8$, (e) $(C_6H_5C_3H_6NH_3)_4AgBiI_8\cdot H_2O$, (f) $(FC_6H_4C_2H_4NH_3)_4AgBiI_8\cdot H_2O$, (g) $(NHC_5H_4C_2H_4NH_3)_2AgBiI_8$, and (h) $(NH_3C_6H_4CH_2NH_3)_2AgBiI_8$.

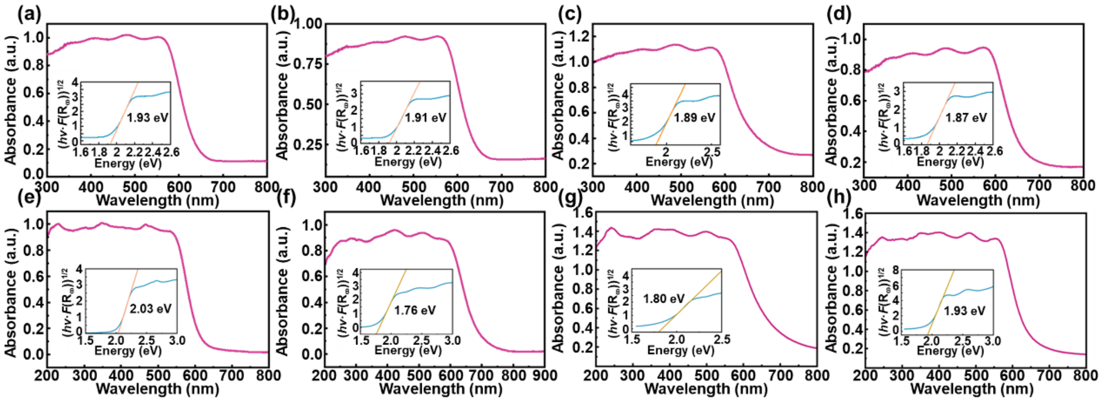

Supplementary Figure 28. UV-vis absorption spectra of synthesized perovskites. UV-vis absorption spectra of (a) $(C_6H_{11}NH_3)_4AgBiI_8$, (b) $(FC_6H_4CH_2NH_3)_4AgBiI_8$, (c) $(ClC_6H_4CH_2NH_3)_4AgBiI_8$, (d) $(BrC_6H_4CH_2NH_3)_4AgBiI_8$, (e) $(C_6H_5C_3H_6NH_3)_4AgBiI_8\cdot H_2O$, (f) $(FC_6H_4C_2H_4NH_3)_4AgBiI_8\cdot H_2O$, (g) $(NHC_5H_4C_2H_4NH_3)_2AgBiI_8$, and (h) $(NH_3C_6H_4CH_2NH_3)_2AgBiI_8$.

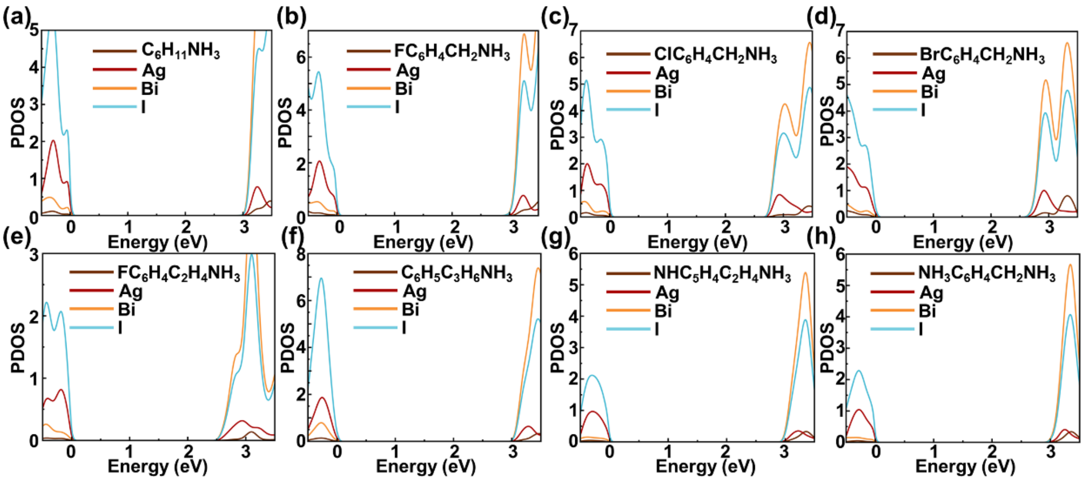

Supplementary Figure 29. DFT-calculated PDOS of synthesized perovskites. DFT-calculated PDOS of (a) $(C_6H_{11}NH_3)_4AgBiI_8$, (b) $(FC_6H_4CH_2NH_3)_4AgBiI_8$, (c) $(ClC_6H_4CH_2NH_3)_4AgBiI_8$, (d) $(BrC_6H_4CH_2NH_3)_4AgBiI_8$, (e) $(FC_6H_4C_2H_4NH_3)_4AgBiI_8$•$H_2O$, (f) $(C_6H_5C_3H_6NH_3)_4AgBiI_8$•$H_2O$, (g) $(NHC_5H_4C_2H_4NH_3)_2AgBiI_8$, and (h) $(NH_3C_6H_4CH_2NH_3)_2AgBiI_8$.

Supplementary Tables

Supplementary Table 1. Crystal data for $(ClC_6H_4CH_4NH_3)_4AgBiI_8$, $(BrC_6H_4CH_4NH_3)_4AgBiI_8$, and $(CH_3C_6H_4C_2H_4NH_3)_4AgBiI_8$.

Compound	$(ClC_6H_4CH_4NH_3)_4$ $AgBiI_8$	$(BrC_6H_4CH_4NH_3)_4$ $AgBiI_8$	$(CH_3C_6H_4C_2H_4NH_3)_4$ $AgBiI_8$
Formula	$C_{32}H_{44}AgBiCl_4I_8N_4$	$C_{32}H_{44}AgBiBr_4I_8N_4$	$C_{36}H_{56}AgBiI_8N_4$
$D_{calc.}$(g cm^{-3})	2.493	2.681	2.368
m (mm^{-1})	8.707	11.403	8.427
Formula Weight	1958.56	2136.40	1876.89
Shape	block-shaped	block-shaped	block-shaped
Size (mm^3)	0.28×0.26×0.24	0.28×0.27×0.26	0.30×0.27×0.24
T (K)	300(2)	293.0	300.30(10)
Crystal System	triclinic	triclinic	monoclinic
Space Group	*P*1	*P*1	$P2_1$
a (Å)	9.048(13)	9.109(5)	9.2212(4)
b (Å)	9.169(12)	9.139(6)	9.0155(3)
c (Å)	15.81(2)	15.977(10)	31.6716(9)
a (˚)	88.79(3)	84.130(20)	90
b (˚)	84.36(3)	89.826(19)	91.009(3)
g (˚)	89.68(2)	89.975(16)	90
V (Å^3)	1305(3)	1323.1(14)	2632.57(16)
Z	1	1	2

Supplementary Table 2. Crystal data for $(ClC_6H_4NH_3)_4AgBiI_8$, $(NH_3C_6H_{10}NH_3)_2AgBiI_8·H_2O$, and $(NH_2C_5H_9CH_2NH_3)_2AgBiI_8·0.5H_2O$.

Compound	$(ClC_6H_4NH_3)_4$ $AgBiI_8$	$(NH_3C_6H_{10}NH_3)_2$ $AgBiI_8·H_2O$	$(NH_2C_5H_9CH_2NH_3)_2$ $AgBiI_8·0.5H_2O$
Formula	$C_{24}H_{28}AgBiCl_4I_8N_4$	$C_{12}H_{34}AgBiI_8N_4O$	$C_{24}H_{66}Ag_2Bi_2I_{16}N_8O$
$D_{calc.}$ (g cm^{-3})	2.784	3.290	3.233
m (mm^{-1})	10.306	13.856	13.693
Formula Weight	1846.35	1582.48	3146.95
Shape	plate-shaped	plate-shaped	prism-shaped
Size (mm^3)	0.31×0.28×0.25	0.25×0.21×0.17	0.30×0.17×0.09
T (K)	300.78(10)	296(2)	296(2)
Crystal System	monoclinic	monoclinic	monoclinic
Space Group	$P2_1/c$	$P2_1/n$	$C2/c$
a (Å)	9.0592(4)	8.556(6)	34.889(15)
b (Å)	8.2493(4)	19.504(15)	8.443(4)
c (Å)	29.6608(19)	19.181(14)	22.116(9)
a (°)	90	90	90.00
b (°)	96.510(4)	93.488(11)	97.109(5)
g (°)	90	90	90.00
V (Å^3)	2202.3(2)	3195(4)	6464(5)
Z	2	4	4

Supplementary Table 3. Crystal data for $(NH_2C_4H_6F_2)_4AgBiI_8{\cdot}H_2O$, $(IC_3H_6NH_3)_4AgBiI_8$, and $(F_3C_4H_6NH_3)_4AgBiI_8$.

Compound	$(NH_2C_4H_6F_2)_4$ $AgBiI_8{\cdot}H_2O$	$(IC_3H_6NH_3)_4$ $AgBiI_8$	$(F_3C_4H_6NH_3)_4$ $AgBiI_8$
Formula	$C_{16}H_{34}AgBiF_8I_8N_4O$	$C_{12}H_{36}AgBiI_{12}N_4$	$C_{16}H_{36}AgBiF_{12}I_8N_4$
$D_{calc.}$ (g cm^{-3})	2.982	3.397	2.740
m (mm^{-1})	11.197	13.948	9.956
Formula Weight	1782.52	2076.10	1844.54
Shape	prism-shaped	block-shaped	plate-shaped
Size (mm^3)	0.20×0.19×0.18	0.22×0.21×0.20	0.23×0.21×0.15
T (K)	303.00	277.00	293(2)
Crystal System	orthorhombic	triclinic	monoclinic
Space Group	*Pbcm*	$P\bar{1}$	$P2_1/c$
a (Å)	8.3967(13)	8.7683(16)	9.5029(5)
b (Å)	20.332(4)	9.4218(15)	8.7165(4)
c (Å)	23.254(4)	12.334(2)	26.993(2)
a (°)	90	90.339(4)	90
b (°)	90	95.111(5)	89.995(6)
g (°)	90	90.212(5)	90
V (Å^3)	3970.0(12)	1014.9(3)	2235.9(2)
Z	4	1	2

Supplementary Table 4. Crystal data for $(ClFC_6H_3NH_3)_4AgBiI_8$, $(NH_2C_5H_9F)_4AgBiI_8{\cdot}H_2O$, and $(NH_2C_5H_8F_2)_4AgBiI_8$.

Compound	$(ClFC_6H_3NH_3)_4$ $AgBiI_8$	$(NH_2C_5H_9F)_4$ $AgBiI_8{\cdot}H_2O$	$(NH_2C_5H_8F_2)_4$ $AgBiI_8$
Formula	$C_{24}H_{24}AgBiCl_4F_4I_8N_4$	$C_{20}H_{46}AgBiF_4I_8N_4O$	$C_{20}H_{40}AgBiF_8I_8N_4$
$D_{calc.}$ (g cm^{-3})	2.838	2.759	2.856
m (mm^{-1})	10.129	10.438	10.501
Formula Weight	1918.32	1766.66	1820.61
Shape	plate-shaped	block-shaped	plate-shaped
Size (mm^3)	0.28×0.26×0.24	0.35×0.26×0.15	0.17×0.15×0.11
T (K)	300.04(10)	299.06(10)	293(2)
Crystal System	monoclinic	monoclinic	monoclinic
Space Group	$P2_1/c$	$P2_1/m$	C_2
a (Å)	8.9716(3)	13.0614(5)	24.4657(7)
b (Å)	8.9460(3)	24.5850(10)	9.2749(3)
c (Å)	28.0828(10)	13.2437(5)	9.3293(3)
a (°)	90	90	90
b (°)	95.191(3)	90.107(4)	90.238(3)
g (°)	90	90	90
V (Å^3)	2244.68(13)	4252.7(3)	2116.95(10)
Z	2	4	2

Supplementary Table 5. Crystal data for $(N_2C_3H_4C_2H_4NH_3)_2AgBiI_8$.

Compound	$(N_2C_3H_4C_2H_4NH_3)_2AgBiI_8$
Formula	$C_{10}H_{20}AgBiI_8N_6$
$D_{calc.}$ (g cm^{-3})	3.340
m (mm^{-1})	14.298
Formula Weight	1556.37
Shape	block-shaped
Size (mm^3)	0.26×0.24×0.22
T (K)	303.00(10)
Crystal System	monoclinic
Space Group	*C*2/*c*
a (Å)	12.3883(3)
b (Å)	12.9473(3)
c (Å)	19.3481(5)
a (°)	90
b (°)	94.113(2)
g (°)	90
V (Å^3)	3095.35(13)
Z	4

Supplementary Table 6. Full list of features created for the synthesis feasibility problem of 2D AgBi iodide perovskites.

Descriptor	Derivation
MolWt	Molecular weight
$Num_{Heteroatoms}$	Number of heteroatoms
$Num_{RotatableBonds}$	Number of rotatable bonds
$Fraction_{CSP3}$	Fraction of carbon Sp^3
$^1\kappa$	The first-order kappa shape index
$^2\kappa$	The second-order kappa shape index
$^3\kappa$	The third-order kappa shape index
$Num_{AromaticCarbocycles}$	Number of aromatic carboncycles
$Num_{AromaticRings}$	Number of aromatic rings
$Num_{AmideBonds}$	Number of amide bonds
$Num_{AtomStereoCenters}$	Number of atom stereo centers
$Num_{BridgeheadAtoms}$	Number of bridgehead atoms
$Num_{SaturatedCarbocycles}$	Number of saturated carboncycles
$Num_{AliphaticCarbocycles}$	Number of aliphatic carboncycles
$Num_{AromaticHeterocycles}$	Number of aromatic heterocycles
$Num_{AliphaticRings}$	Number of aliphatic rings
$Num_{LipinskiHBA}$	Number of Lipinski hydrogen bond acceptors
$Num_{LipinskiHBD}$	Number of Lipinski hydrogen bond donors
Num_{Rings}	Number of rings
$Num_{SaturatedHeterocycles}$	Number of saturated heterocycles
$Num_{SaturatedRings}$	Number of saturated rings
$Num_{SpiroAtoms}$	Number of spiro atoms
$Num_{UnspecifiedAtomStereoCenters}$	Number of unspecified atom stereo centers
$Num_{Heterocycles}$	Number of heterocycles
E_{HOMO}	Energy of the highest occupied molecular orbital
E_{LUMO}	Energy of the lowest occupied molecular orbital
E_{HOMO}-E_{LUMO}	Energy gap between E_{HOMO} and E_{LUMO}
Dipole	Dipole of molecules
x	Length of molecules
y	Width of molecules
z	Height of molecules
STEI	Steric effect index of nitrogen
DIS_{NN}	Distance between two nitrogen atoms
Eccentricity	The maximum distance between nitrogen and other atoms
Num_{Rot}	Number of rotatable bonds on the tail of molecules
Num_N	Number of nitrogen atoms

Supplementary Table 7. Bond length, penetration depth, and modulus of PbI_4 and $AgBiI_8$.

	PbI_4	$AgBiI_8$
Metal-iodide bond length (Å)	3.293	3.060
Metal-metal bond length (Å)	6.098	6.038
Penetration depth (Å)	0.546	0.526
Modulus (GPa)	20.433	56.960

Supplementary Table 8. Alpha value from covalent radii.[20]

Atom valence state	r (Å)	α
C (sp^3)	0.77	0.0
C (sp^2)	0.67	-0.13
C (sp)	0.60	-0.22
N (sp^3)	0.74	-0.04
N (sp^2)	0.62	-0.20
N (sp)	0.55	-0.29
O (sp^3)	0.74	-0.04
O (sp^2)	0.62	-0.20
F	0.72	-0.07
P (sp^3)	1.10	0.43
P (sp^2)	1.00	0.30
S (sp^3)	1.04	0.35
S (sp^2)	0.94	0.22
Cl	0.99	0.29
Br	1.14	0.48
I	1.33	0.73

Supplementary Table 9. Feature coefficients of equations obtained from trained SVC model.

System[a]	Dis_{NN}	STEI	Eccentricity	Num_N	Num_{Rot}	MolWt	C
**1**	-1.981	-2.249	-1.037	-1.581	2.158	-0.032	14.007
**2**	-6.985	-2.722	-1.788	-0.956	2.615	-0.037	17.353
**3**	-1.981	-2.249	-1.037	-1.581	2.158	-0.032	14.007
**5**	-1.981	-2.249	-1.037	-1.581	2.158	-0.032	14.007
**10**	-2.236	-1.092	-0.144	0.014	1.043	-0.006	2.925
**11**	-2.158	-2.052	-1.546	-1.452	2.814	-0.023	13.435
**12**	-2.302	-1.114	-0.176	0.003	1.074	-0.007	3.159
**24**	-4.131	-4.595	-3.221	-1.895	4.417	-0.037	27.077
**27**	-10.971	-2.529	-1.751	-0.997	3.045	-0.039	17.184
**28**	-5.286	-2.481	-1.184	-1.793	2.371	-0.037	15.965
**29**	-12.803	-2.447	-2.246	-0.528	3.184	-0.018	15.983
**30**	-3.486	-3.804	-0.811	-3.453	3.358	-0.037	19.542
**32**	-16.173	-1.123	-1.486	-0.296	3.357	-0.034	11.814
**33**	-1.981	-2.249	-1.037	-1.581	2.158	-0.032	14.007
**45**	-1.981	-2.249	-1.037	-1.581	2.158	-0.032	14.007
**49**	-2.076	-2.551	-1.768	-1.413	3.108	-0.040	17.837
**50**	-5.003	-2.561	-1.431	-1.718	2.702	-0.037	16.788
**53**	-1.981	-2.249	-1.037	-1.581	2.158	-0.032	14.007
**54**	-1.908	-2.436	-1.251	-1.747	2.333	-0.034	15.659
**57**	-5.003	-2.561	-1.431	-1.718	2.702	-0.037	16.788

[a] System represents the sample took out, which includes CC(C1=CC=C(C=C1)Cl)N (**1**), CC(C1=CC=C(C=C1)Br)N (**2**), CC1=CC=C(C=C1)C(C)N (**3**), C1=CC(=CC=C1N)Cl (**5**), C1=CC(=CN=C1)Br (**10**), CC(C)C1=CC=C(C=C1)N (**11**), CN(C)C1=CC=CC=C1 (**12**), C1=CC(=CN=C1)I (**24**), C1CC(CNC1)F (**27**), C1CC(CCC1N)N (**28**), C1CNCC1(F)F (**29**), C1CNCCC1(F)F (**30**), C1CNCCC1CN (**32**), C1=C(NC=N1)CCN (**33**), CN(C)C1CCNC1 (**45**), C1CCNC(C1)N (**49**), C1CNC(CN1)O (**50**)， C1CCN(CC1)N (**53**), C1CSCCN1 (**54**), C1CS(=O)(=O)CCN1 (**57**).

Supplementary Table 10. Crystal data for $(C_6H_{11}NH_3)_4AgBiI_8$ and $(FC_6H_4CH_2NH_3)_4AgBiI_8$.

Compound	$(C_6H_{11}NH_3)_4AgBiI_8$	$(FC_6H_4CH_2NH_3)_4AgBiI_8$
Formula	$C_{24}H_{56}AgBiI_8N_4$	$C_{28}H_{36}AgBiF_4I_8N_4$
$D_{calc.}$ (g cm^{-3})	2.595	2.722
m (mm^{-1})	9.991	9.909
Formula Weight	1732.77	1836.66
Shape	block-shaped	plate-shaped
Size (mm^3)	0.30×0.29×0.28	0.26×0.25×0.24
T (K)	300(2)	293(2)
Crystal System	monoclinic	monoclinic
Space Group	$P2_1/c$	*Pn*
a (Å)	9.346(2)	8.7637(12)
b (Å)	8.946(2)	9.6561(13)
c (Å)	26.529(5)	26.802(3)
a (°)	90.00(3)	90
b (°)	90.51(3)	98.858(4)
g (°)	90.00(3)	90
V (Å^3)	2217.9(8)	2241.0(5)
Z	2	2

Supplementary Table 11. Crystal data for $(ClC_6H_4CH_2NH_3)_4AgBiI_8$ and $(BrC_6H_4CH_2NH_3)_4AgBiI_8$.

Compound	$(ClC_6H_4CH_2NH_3)_4AgBiI_8$	$(BrC_6H_4CH_2NH_3)_4AgBiI_8$
Formula	$C_{28}H_{36}AgBiCl_4I_8N_4$	$C_{28}H_{36}AgBiBr_4I_8N_4$
$D_{calc.}$ (g cm^{-3})	2.699	2.868
m (mm^{-1})	9.700	12.522
Formula Weight	1902.46	2080.30
Shape	plate-shaped	plate-shaped
Size (mm^3)	0.28×0.27×0.26	0.32×0.23×0.20
T (K)	269.00	300.80(10)
Crystal System	monoclinic	monoclinic
Space Group	$P2_1/n$	$P2_1/n$
a (Å)	8.662(4)	8.7592(5)
b (Å)	8.964(5)	9.0061(5)
c (Å)	30.264(17)	30.6490(16)
a (°)	90	90
b (°)	95.018(8)	94.919(5)
g (°)	90	90
V (Å^3)	2341(2)	2408.9(2)
Z	2	2

Supplementary Table 12. Crystal data for $(C_6H_5C_3H_6NH_3)_4AgBiI_8{\cdot}H_2O$ and $(FC_6H_4C_2H_4NH_3)_4AgBiI_8{\cdot}H_2O$.

Compound	$(C_6H_5C_3H_6NH_3)_4AgBiI_8{\cdot}H_2O$	$(FC_6H_4C_2H_4NH_3)AgBiI_8{\cdot}H_2O$
Formula	$C_{36}H_{58}AgBiI_8N_4O$	$C_{32}H_{42}AgBiF_4I_8N_4O$
$D_{calc.}$ (g cm^{-3})	2.310	2.618
m (mm^{-1})	8.147	9.186
Formula Weight	1894.91	1906.74
Shape	plate-shaped	plate-shaped
T (K)	288.00	293.00
Crystal System	monoclinic	triclinic
Space Group	$P2_1$	*P-1*
a (Å)	9.727(5)	8.622(11)
b (Å)	17.232(6)	8.722(11)
c (Å)	16.685(7)	16.28(2)
a (°)	90	98.90(2)
b (°)	103.097(10)	89.82(3)
g (°)	90	89.987(19)
V (Å^3)	2724(2)	1210(3)
Z	2	1

Supplementary Table 13. Crystal data for $(NHC_5H_4C_2H_4NH_3)_2AgBiI_8$ and $(NH_3C_6H_4CH_2NH_3)_2AgBiI_8$.

Compound	$(NHC_5H_4C_2H_4NH_3)_2AgBiI_8$	$(NH_3C_6H_4CH_2NH_3)_2AgBiI_8$
Formula	$C_7H_{11}Ag_{0.48}Bi_{0.52}I_4N_2$	$C_{14}H_{24}AgBiI_8N_4$
$D_{calc.}$ (g cm^{-3})	3.300	3.247
m (mm^{-1})	14.074	13.691
Formula Weight	790.97	1580.42
Shape	block-shaped	block-shaped
T (K)	283.00	305.00
Crystal System	monoclinic	triclinic
Space Group	*P*2/*c*	*P-1*
a (Å)	12.737(2)	8.4382(11)
b (Å)	6.3462(12)	9.1645(14)
c (Å)	19.873(4)	11.3224(16)
a (°)	90	109.114(4)
b (°)	97.704(5)	100.712(4)
g (°)	90	92.213(4)
V (Å^3)	1591.8(5)	808.2(2)
Z	4	1

Supplementary Table 14. Validation results of ML models based on the various methods for imbalanced dataset. ML^{SMOTE}, ML^{Con}, and ML^{EEC} represents SMOTE, CondensedNearestNeighbour, and EasyEnsembleClassifier method, respectively.

Compounds	Test	ML^{SMOTE}	ML^{Con}	ML^{EEC}	$ML^{our\ work}$
$(C_5H_{11}N_3)_2AgBiI_8$	2D	Non-2D	Non-2D	2D	2D
$(C_6H_7NCl)_4AgBiI_8$	2D	2D	2D	2D	2D
$(C_4H_9NF_3)_4AgBiI_8$	2D	Non-2D	2D	2D	Non-2D
$(C_8H_{20}N_2)_2AgBiI_8$	Non-2D	2D	2D	2D	Non-2D
$(C_8H_{11}NBr)_4AgBiI_8$-1	Non-2D	2D	2D	2D	Non-2D
$(C_8H_{11}NBr)_4AgBiI_8$-2	Non-2D	2D	2D	2D	Non-2D
$(C_5H_8NS)_4AgBiI_8$	Non-2D	Non-2D	2D	2D	Non-2D
$(C_5H_5NI)_4AgBiI_8$	Non-2D	2D	2D	2D	2D
$(C_2H_7NBr)_4AgBiI_8$	Non-2D	Non-2D	2D	2D	Non-2D
$(C_5H_5NBr)_4AgBiI_8$	Non-2D	2D	2D	2D	2D

Supplementary References

1. Dolomanov, O. V., Bourhis, L. J., Gildea, R. J., Howard, J. A. K. & Puschmann, H. OLEX2: a complete structure solution, refinement and analysis program. *J. Appl. Cryst.* **42,** 339-341 (2009).
2. Sheldrick, G. M. SHELXT - integrated space-group and crystal-structure determination. *Acta Cryst.* **A71,** 3-8, (2015).
3. Sheldrick, G. M. Crystal structure refinement with SHELXL. *Acta Cryst.* **C71,** 3-8, (2015).
4. Lavrac N., Kavsek B., Flach P., et al. Subgroup discovery with CN2-SD. *J. Mach. Learn. Res.* **5,** 153-188 (2004).
5. Randic, M. Characterization of molecular branching. *J. Am. Chem. Soc.* **97,** 6609-6615 (1975).
6. Lyu, R., Moore, C. E., Liu, T., Yu, Y. & Wu, Y. Predictive design model for low-dimensional organic-inorganic halide perovskites assisted by machine learning. *J. Am. Chem. Soc.* **143,** 12766-12776 (2021).
7. Kamminga, M. E. et al. Confinement effects in low-dimensional lead iodide perovskite hybrids. *Chem. Mater.* **28,** 4554-4562 (2016).
8. Yin, Z. & Hou, J. Recent advances on SVM based fault diagnosis and process monitoring in complicated industrial processes. *Neurocomputing* **174,** 643-650 (2016).
9. Wang, V. Xu, N. Liu, J.C. Tang, G. Geng, W.T. VASPKIT: A user-friendly interface facilitating high-throughput computing and analysis using VASP code, *Comput. Phys. Commun.* **267,** 108033 (2021)
10. Maaten, L. v. d. & Hinton, G. Visualizing data using t-SNE. *J. Mach. Learn. Res.* **9,** 2579-2605 (2008).
11. Kresse, G. & Furthmuller, J. Efficiency of ab-initio total energy calculations for metals and semiconductors using a plane-wave basis set. *Comput. Phys. Sci.* **6,** 15-50 (1996).
12. Blöchl, P. E. Projector augmented-wave method. *Phys. Rev. B* **50,** 17953-17979 (1994).

13. Perdew, J. P., Burke, K. & Ernzerhof, M. Generalized gradient approximation made simple. *Phys. Rev. Lett.* **77,** 3865 (1996).

14. Heyd, J., Peralta, J. E., Scuseria, G. E. & Martin, R. L. Energy band gaps and lattice parameters evaluated with the Heyd-Scuseria-Ernzerhof screened hybrid functional. *J. Chem. Phys.* **123,** 174101 (2005).

15. Heyd, J., Scuseria, G. E. & Ernzerhof, M. Hybrid functionals based on a screened Coulomb potential. *J. Chem. Phys.* **118,** 8207-8215 (2003).

16. Grimme, S., Antony, J., Ehrlich, S. & Krieg, H. A consistent and accurate ab initio parametrization of density functional dispersion correction (DFT-D) for the 94 elements H-Pu. *J. Chem. Phys.* **132,** 154104 (2010).

17. Momma, K. & Izumi, F. VESTA: a three-dimensional visualization system for electronic and structural analysis. *J. Appl. Crystallogr.* **41,** 653–658 (2008).

18. Mitzi, D. B. A layered solution crystal growth technique and the crystal structure of $(C_6H_5C_2NH_3)_2PbCl_4$. *J. Solid State Chem.* **145,** 694–704 (1999).

19. Xu, Z. et al. A lead-free I-based hybrid double perovskite $(I\text{-}C_4H_8NH_3)_4AgBiI_8$ for X-ray detection. *J. Mater. Chem. C* **9,** 13157-13161 (2021).

20. Hall, L. H. & Kier, L.B. The molecular connectivity chi indexes and kappa shape indexes in structure-property modeling. *Rev. Comput. Chem.* 367–422 (1991).
