# Supplementary material for: Universal machine learning aided synthesis approach of two-dimensional perovskites in a typical laboratory
Source: Nat Commun. 2024 Jan 2;15:138. doi: 10.1038/s41467-023-44236-5 (PMC10761762; doi:10.1038/s41467-023-44236-5)
Supplement: Supplementary file 3 — Description of Additional Supplementary Files [file 41467_2023_44236_MOESM3_ESM.docx]

**Description of Additional Supplementary Files**

**Supplementary Data 1:** Crystallographic Information Files of synthesized perovskites.

**Supplementary Data 2:** Visualization of prediction results of all compounds in the prediction set.
